# Effects on community composition and function *Pinus massoniana* infected by *Bursaphelenchus xylophilus*

**DOI:** 10.1186/s12866-022-02569-z

**Published:** 2022-06-11

**Authors:** Xin Hao, Xuefeng Liu, Jie Chen, Bowen Wang, Yang Li, Yi Ye, Wei Ma, Ling Ma

**Affiliations:** 1 Northeast Forestry University, Harbin, China; 2 Present Address: Wageningen University & Research, Wageningen, Netherlands; 3 Zhejiang University, Hangzhou, China; 4 Heilongjiang University of Chinese Medicine, Harbin, China

**Keywords:** Microbial community structure, *Pinus massoniana*, Illumina MiSeq sequence, Diversity analysis, Function analyzed

## Abstract

Pine wilt disease (PWD) is a worldwide forest disease caused by pine wood nematode (PWN). In this article, we investigated the composition, organization, correlation, and function of the endophytic microbial community in *Pinus massoniana* field with and without PWN. Samples were taken from branches, upper, middle, and lower trunks, as well as soil, from both healthy and infected trees. The results showed that the fungal diversity of healthy pines is around 1.1 times that of infected pines, while the bacterial diversity is about 0.75 times that of infected pines at the OTUs level. An increase of the abundance of pathogenic fungus such as *Saitozyma*, *Graphilbum*, *Diplodia*, *Candida*, *Pseudoxanthomonas*, *Dyella* and *Pantoea* was witnessed in infected pines according to the result of LEfSe. Furthermore, *Ophiostoma* and saprophytic fungus such as *Entomocorticium, ganoderma, tomentella, entomocorticium* were exclusively prominent in infected pines, which were substantially and highly connected with other species (*p* < 0.05), indicating the trees’ vulnerability and making the wood blue. In healthy pines, the top three functional guilds are parasites, plant pathogens, and saprotrophs. Parasites (36.52%) are primarily found in the branches, plant pathogens (29.12%) are primarily found in the lower trunk, and saprotrophs (67.88%) are primarily found in the upper trunk of disease trees. Pines’ immunity is being eroded due to an increase in the quantity and types of diseases. PICRUSt2 research revealed that NADH or NADPH, as well as carbon-nitrogen bonds, were more abundant in healthy pines, but acid anhydrides and transferring phosphorus-containing groups were more abundant in infected pines. The shift in resin secretion lowers the tree’s potential and encourages pine wilt and mortality. In total, PWN may have disrupted the microbiological ecology and worked with the community to hasten the demise of pines.

**Supplementary Information:**

The online version contains supplementary material available at 10.1186/s12866-022-02569-z.

## Introduction

Pine wilt disease (PWD) is a devastating forest disease that has struck east Asia and portions of Europe [1, 2]. The disease has expanded over China’s eastern and western regions in recent years, causing massive economic losses and environmental dangers. *Bursaphelenchus xylophilus*, a pathogen, has been identified as one of China’s most harmful forestry organisms. The pathogenic mechanism of the pinewood nematode (PWN) has aroused widely concern of researchers. There are several theories on how PWN causes disease, including the cellulase theory, phytotoxin hypothesis, and terpenoid hypothesis [3]. According to the cellulase theory, PWN release cellulase to damage pine parenchyma cells, causing pine wilt [4–6]. The phytotoxin theory states that PWN invades the pine tree and produces or causes a poisonous chemical that disrupts the pine tree’s metabolism and causes it to wilt [7, 8]. The both hypotheses have limitations that they fail to explain why larger pines wilt earlier than smaller pines after being infected with PWN [9]. The terpenoid hypothesis illustrated that PWN affects the transfer of water and nutrients in pine trees, which causes needle wilt and tree mortality [10]. The aforementioned hypotheses are extremely important in determining the pathogenic mechanism of PWN. However, these investigations concentrate on a few visible events in the pathogenic process of PWN. They are unable to adequately reflect the disease’s pathogenic mechanism.

Recent research has discovered that both PWN infection and host insect invasion can alter the microbial community in pines’ internal and exterior environments, and the changing of the microbial community is a critical component in the prevalence of PWD [11, 12]. It was discovered that Enterobacter dominated the gut microbial population of *Monochamus alternatus* larvae. These bacteria may aid *M. alternatus* in the breakdown of cellulose and pinene, according to macro-genomic research [13]. Previous study has shown that PWN infection in *Pinus thunbergia* can alter the microbial community structure and nutritional composition of the rhizosphere soil [14]. The quantity of endophytic bacteria in *P. pinaster* altered after PWN infection [15]. In *P. thunbergii*, PWN infection altered the rhizosphere and needle microbial communities [16, 17]. In *P. massoniana*, PWN infection altered the forest soil characteristics and rhizosphere microbial community [18]. In addition, Sriwati et al. discovered that some fungus can help *P. thunbergii* reproduce PWN [19]. The presence of PWD impacted the rhizosphere soil characteristics in several types of pines and modified the microbial community makeup of pine tissues, according to various studies. As a result, research into microbial populations in tree trunks is required.

In this study, we collected samples of branches, trunks, and rhizosphere soil from *P. massoniana* infected by PWN in the field. The makeup of microbial communities, structure, correlation and function in healthy and diseased trees was then studied. The study focuses on the function and correlation of the microbial community in PWN-infested pines, particularly the relationship between fungus and bacteria. On this basis, the features of microbial diversity in each area of the pines during PWN invasion and the features of microbial diversity of each section of the pines during PWN invasion were discussed in further depth. On this basis, the features of microbial diversity of each section of the pines during PWN invasion were discussed in further detail. It suggests that PWN infection hastened the mortality of pines by changing the endophytic community structure, providing a theoretical basis for developing PWN management approaches.

## Materials and methods

### Samples sites and collection

All samples were collected in Tianmu Mountain, Lin’an, Zhejiang Province, China (30°20′N, 119°25′E). The environmental characteristics of this region are as following: average annual sunshine hour of 1920, a sunshine rate of 44%, an average annual temperature of 16 °C, an average annual precipitation of 1613.9 mm, an average annual precipitation time of 158 days, an average annual frost-free period of 237 days, and a total area 3118.77 km^2^. *P. massoniana* and *Cunninghamia lanceolata* make up the majority of the trees in this stand. We chose three healthy trees and three diseased trees infected exclusively with PWN as samples, following Millberg’s methodology [20]. Healthy trees have entirely green needles. On the contrary, diseased trees have reddish-brown needles that do not fall off owing to PWN infection, bark with longicorn beetle nests, the trunk turns blue, and no turpentine loss signs. Secondary branches, trunks (upper, middle, and lower), surface soil (0-5 cm), and deep soil (5-15 cm) (551 cm) were all sampled from healthy and sick *P. massoniana*. After collection, all samples were immediately deposited in solid carbon dioxide (dry ice), then returned to the laboratory and stored at − 80 °C until required.

### DNA extraction, ITS region amplification and Illumina-MiSeq sequencing

The CTAB technique (Cetyltrimethylammonium Bromide) was used to extract genomic DNA from all of the materials (trees and soil) [21]. First, the trees samples (0.3 g) and soil samples (0.1 g) were frozen with liquid nitrogen. Second, 500 μL DNA extraction buffer (100 mM Tris HCl [pH 8.0], 20 mM EDTA [C_10_H_14_N_2_Na_2_O_8_, pH 8.0], 10% SDS (C_12_H_25_SO_4_Na), 1.4 M NaCl and 2% CTAB) were added in each of samples, treated in 60 °C water bath for 30 mins. Then, the samples were mixed with 200 μL chloroform-isopentenyl liquid (24:1, v/v). The supernatant was precipitated with 0.9 volume isopropanol at room temperature for 20 minutes, centrifuged at 20000 g for 30 minutes, washed twice with 70% cold ethanol, and then dissolved with 50 μL RNase-free water after spinning at 20000 g for 30 minutes. The NanoDrop 2000 ultraviolet spectrophotometer was used to measure the concentrations of the samples (Thermo Fisher Scientific). All extracted DNA samples be stored at − 20 °C until the following step.

The ITS1 region of the fungal ITS gene was amplified using primer combinations ITS1F/ITS2R (ITS1F: 5′-CTTGGTCATTTAGAGGAAGTAA-3′, ITS2R: 5′-GCTGCGTTCTTCATCGATGC-3′) [22] and 338F/806R (338F: 5′-ACTCCTACGGGAGGCAGCAG-3′, 806R: 5′-GGACTACHVGGGTWTCTAAT-3′) [23]. TransStart Fastpfu DNA Polymerase (TransStart) was used in all PCR experiments. The PCR amplifiers were purified by AxyPrep DNA kit (Axygen Biosciences, Central Avenue, Union City, CA, USA), and the PCR products were quantitatively detected by QuantiFluor™ -ST blue fluorescence quantitative system (Promega). The DNA concentration was adjusted to 10 ng/L, and the extracted DNA quality was assessed using a NanoDrop 2000 ultraviolet spectrophotometer and agarose gel electrophoresis. Amplicon library sequencing is performed on the Illumina MiSeq PE300 platform (Illumina, San Diego, CA, USA) using the Majorbio Technology, Shanghai, China, standard methodology. The clean reads may be obtained in the Sequence Read Archive (SRA) repository at the National Center for Biotechnology Information (https://submit.ncbi.nlm.nih.gov/subs/sra/), with the accession number PRJNA 768116.

### Sequence data analysis, Illumina MiSeq data information, and statistical analysis

We screened the raw data, processed it, and quality-controlled it to acquire high-quality and effective tags, which improved the accuracy and reliability of the findings [24]. To begin, the FLASH program (https://ccb.jhu.edu/software/FLASH/) is used to splice the PE reads produced by the initial double-terminal sequencing [25]. Simultaneously, the Fastp program (https://github.com/OpenGene/fastp/) is used to check the quality of the original sequence, differentiate samples by barcode, and eliminate low-quality sequences.

The operation taxon (operational taxonomic units, OUTs) was grouped using the UPARSE algorithm [26] and Usearch software (version 7.0 http://drive5.com/usearch/), and chimeric was removed based on a 97% similarity threshold [27]. The UNITE database is then used to compare each sequence (Release 6.0 http://unite.ut.ee/index.php) [28]. To obtain the species categorization annotation results, the alignment threshold was set to 70% and non-fungal sequences were eliminated from the OTUs (operational taxonomic units). QIIME [29], Mothur [30] and R software were used to examine OTUs abundance, alpha diversity, beta diversity, and community outcomes of species at each categorization level. The makeup of the microbial community structure was then shown in the same way [31, 32].

### Microbial community composition, organization, correlation, and function analysis

To compare the community richness (Richness and Chao1), diversity (Shannon), and evenness (Shannon even) of diseased and healthy trees, one-way analysis of variance (ANOVA) tests was utilized. With InteractiVenn (http://www.interactivenn.net), Venn diagrams were created using subsampled data to reveal common and unique OTUs [33]. The microbial taxonomic and functional groups differentially represented across treatments were identified using linear discriminant analysis (LDA) combined with effect size (LEfSe; http://huttenhower.sph.harvard.edu/galaxy/root?tool id = PICRUSt normalize) [34]. LDA > 4.8 with *p* < 0.05 was used as the LEfSe criterion. The fungal community structure was shown using principal coordinates analysis (PCoA).

The top 50 genera in terms of total genus abundance were used to calculate the correlation between the abundance of species in healthy and diseased trees using Spearman’s correlation algorithm (https://cran.r-project.org) with absolute value of Spearman correlation > 0.5 and false discovery rate-corrected (p < 0.05) to better understand the role and correlation of important microbial genera in the pathogenesis of PWN. Cytoscape 3.7.1 was used to view the networks. The SPLS (Sparse Partial Least Squares) method was used to determine the relationship between fungal and bacteria in healthy and diseased trees. SPLS was written in R (https://cran.r-project.org/web/packages/spls/), and the R-package was available for download via CRAN (the Comprehensive R Archive Network) (https://cran.r-project.org/index.html) [35]. The circus (http://www.circos.ca/) displayed the microbiome divergence.

The COG family information and KEGG Orthology (KO) information corresponding to OTUs were obtained by the Greengene id, corresponding to each OUTs, and the abundance and KO abundance of each COG were calculated [36]. The description information of each COG and its function information may be parsed from the eggNOG database using the COG database’s information, and therefore the function abundance spectrum can be derived. The abundance of each functional category can be determined using the information from the KEGG database, and the abundance of each functional category may be computed using the abundance of OTUs [37]. FUNGuild (Fungi Functional Guild) was utilized to forecast the probable activities of fungi by using bioinformatics approaches to combine fungal species categorization with functional guild classification [38].

Microsoft Office was used to arrange the data, and Student’s t-tests and ANOVA in SPSS 22.0 were used to look for significant differences. Statistical significance was determined for all comparisons using a *p* < 0.05.

## Results

### Microbial composition of diseased and healthy trees

**Fig. 1:**
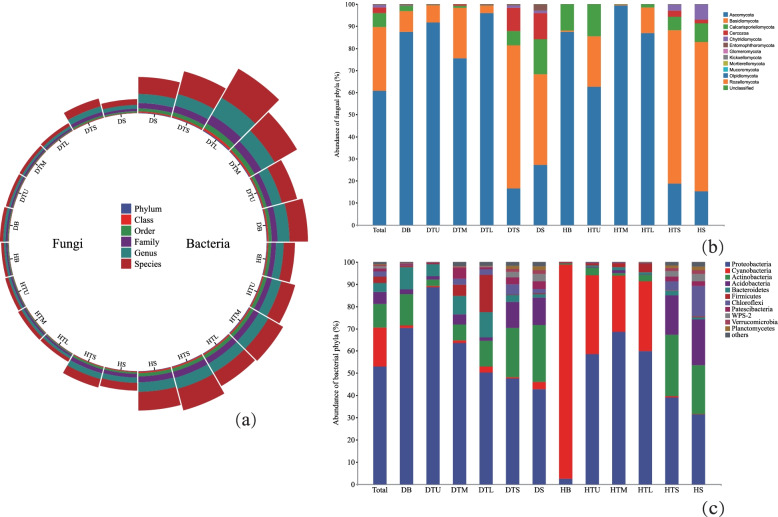
The relative abundance of microbial phyla (as a percentage of total reads) in healthy and sick samples of *P. massoniana* branches, trunks, and soil. Ranches are represented by HB and DB, upper trunk by HTU and DTU, middle trunk by HTM and DTM, lower trunk by HTL and DTL, surface soil by HTS and DTS, and deep soil by HS and DS. Others means the relative abundance of microbial phyla below 1%

A total of 3,601,568 (fungi) and 2,240,660 (bacteria) high-quality sequences were generated across all samples after sequence de-noising and quality filtering. The number of fungal communities was less in diseased trees than in healthy trees at all classification levels, while bacteria communities were opposite (Fig. 1a, Table S1). The analysis of the α diversity index of diseased trees and healthy trees at the OTUs level showed that there was no significant difference in microbial community richness and evenness between diseased trees and healthy trees, but the fungal diversity index was a significant difference. In addition, the community richness of healthy and diseased trees was the highest, followed by branches and trunks (Table S2).

All sequences were classified to the fungal domain and assigned to 2830 OTUs across all samples, including 14 phyla, 46 classes, 109 orders, 454 genera and 679 species. Ascomycota (59.80% of the sequence) was the most abundant phylum, followed by Basidiomycota (29.90% of the sequence), Mortierellomycota (2.63% of the sequence), Rozellomycota (1.11% of the sequence), and others (< 1.0% of the sequence) include Mucoromycota, Glomeromycota, Chytridiomycota, Entomophthoromycota, Calcarisporiellomycota, Cercozoa, Kickxellomycota, Olpidiomycota (Fig. 1b, Table S3). All sequences were classified to the bacterial domain and assigned to 6720 OTUs across all samples, including 51 phyla, 114 classes, 315 orders, 565 families, 1280 genera and 2546 species. Proteobacteria (52.87% of the sequence) was the most abundant phylum, followed by Cyanobacteria (17.71% of the sequence), Actinobacteria (10.57% of the sequence), Acidobacteria (5.31% of the sequence), Bacteroidetes (4.14% of the sequence), Firmicutes (2.86% of the sequence), Chloroflexi (2.26% of the sequence), Patescibacteria (1.48% of the sequence) and others (< 1.0% of the sequence) include WPS-2, Verrucomicrobia, Planctomycetes and so on (Fig. 1c, Table S4).

### Microbial structure of diseased and healthy trees

**Fig. 2:**
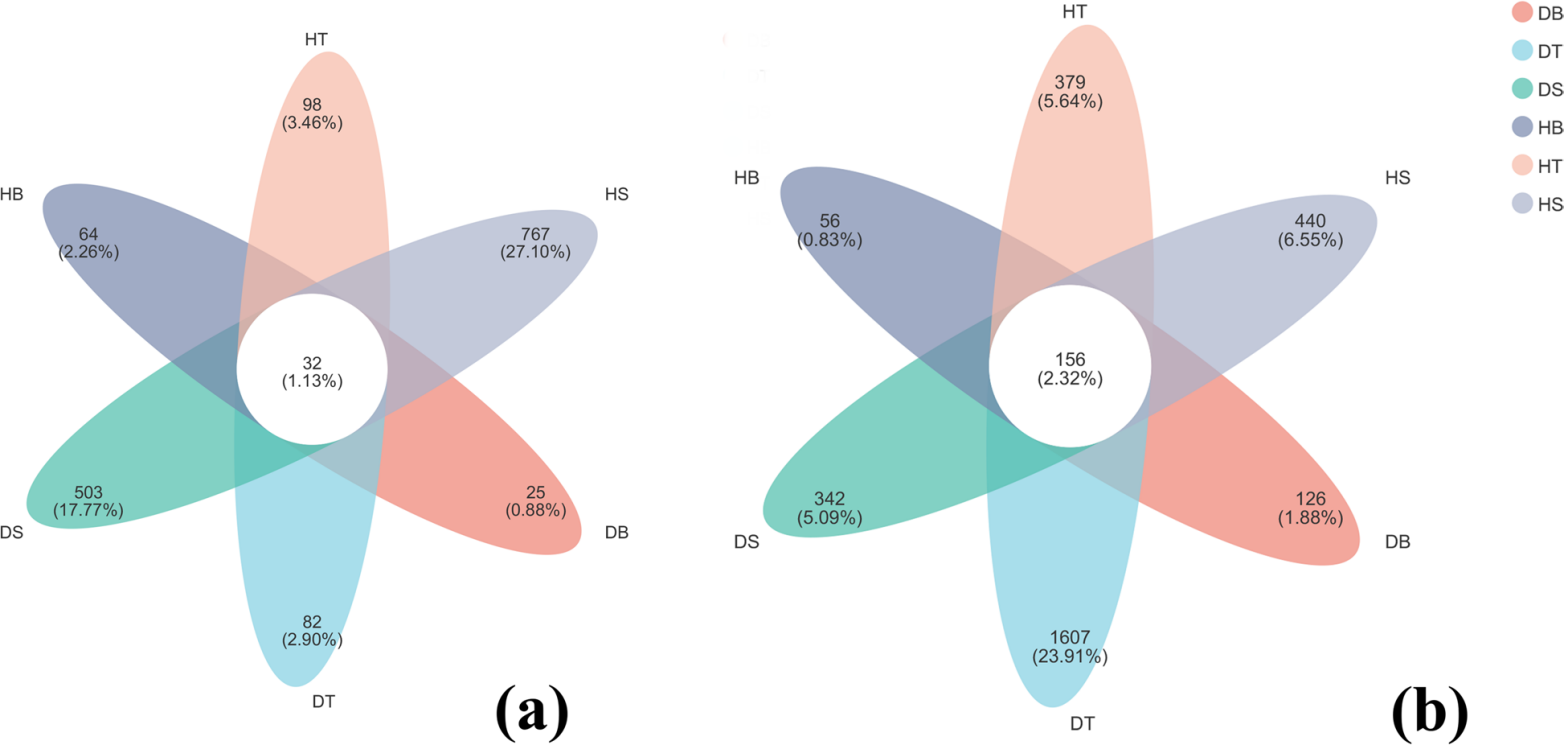
The unique and common OTUs between healthy and diseased trees are depicted in a Venn diagram. Branches, trunks and soil of healthy and diseased trees are represented by HB, HT, HS, DB, DT and DS, respectively

In the analysis of the fungal community of diseased and healthy trees, it was found that the number of unique OTUs in branches, trunks and soil of healthy samples was more than that of diseased samples. The soil shared 40.30% of the OTUs (surface soil 37.34% and the deep soil 29.96%) between healthy and diseased samples, followed by trunks and branches. Only 1.13% of OTUs were shared in branches, trunks and soil, in which the soil harbored the most abundant OTUs, followed by branches and trunks (Fig. 2a). In the analysis of the bacterial community of diseased trees and healthy trees, it was found that the number of unique OTUs in branches, trunks and soil of healthy samples was more than that of diseased samples. The trunks shared 63.90% of the OTUs (upper trunk 21.64%, middle trunk 35.51%, lower trunk 41.37%) between healthy and diseased samples, followed by the soil and branches. Only 2.32% of OTUs were shared in branches, trunks and soil, of which OTUs, in which the trunks harbored the most abundant OTUs, followed by soil and branches (Fig. 2b).

**Fig. 3:**
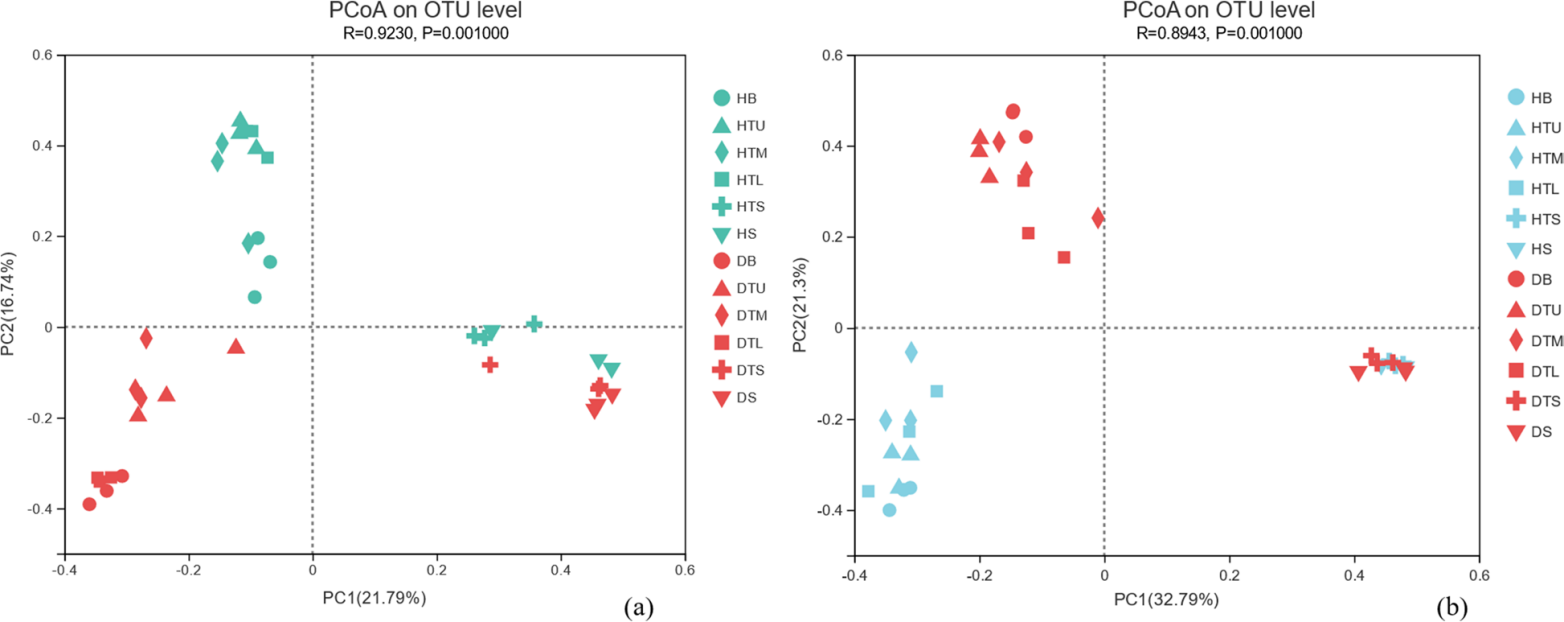
The microbial community structure in healthy and sick samples in the branches, trunks, and soil is shown using principal coordinates analysis (PCoA) based on Bray-Curtis distance. Branches are represented by HB and DB, upper trunk by HTU and DTU, middle trunk by HTM and DTM, lower trunk by HTL and DTL, surface soil by HTS and DTS, and deep soil by HS and DS

PCoA analysis among fungal communities was performed based on Bray-Curtis distance with the first and second axes explaining 21.79 and 16.74% of the variance, respectively (Fig. 3a). PCoA analysis among bacterial communities was performed based on Bray-Curtis distance with the first and second axes explaining 32.79 and 21.3% of the variance, respectively (Fig. 3b). The results showed that the infection of PWN mainly affected the endophytic microbial community of branches and trunks of *P. massoniana*, but had little effect on the microbial community in soil.

**Fig. 4:**
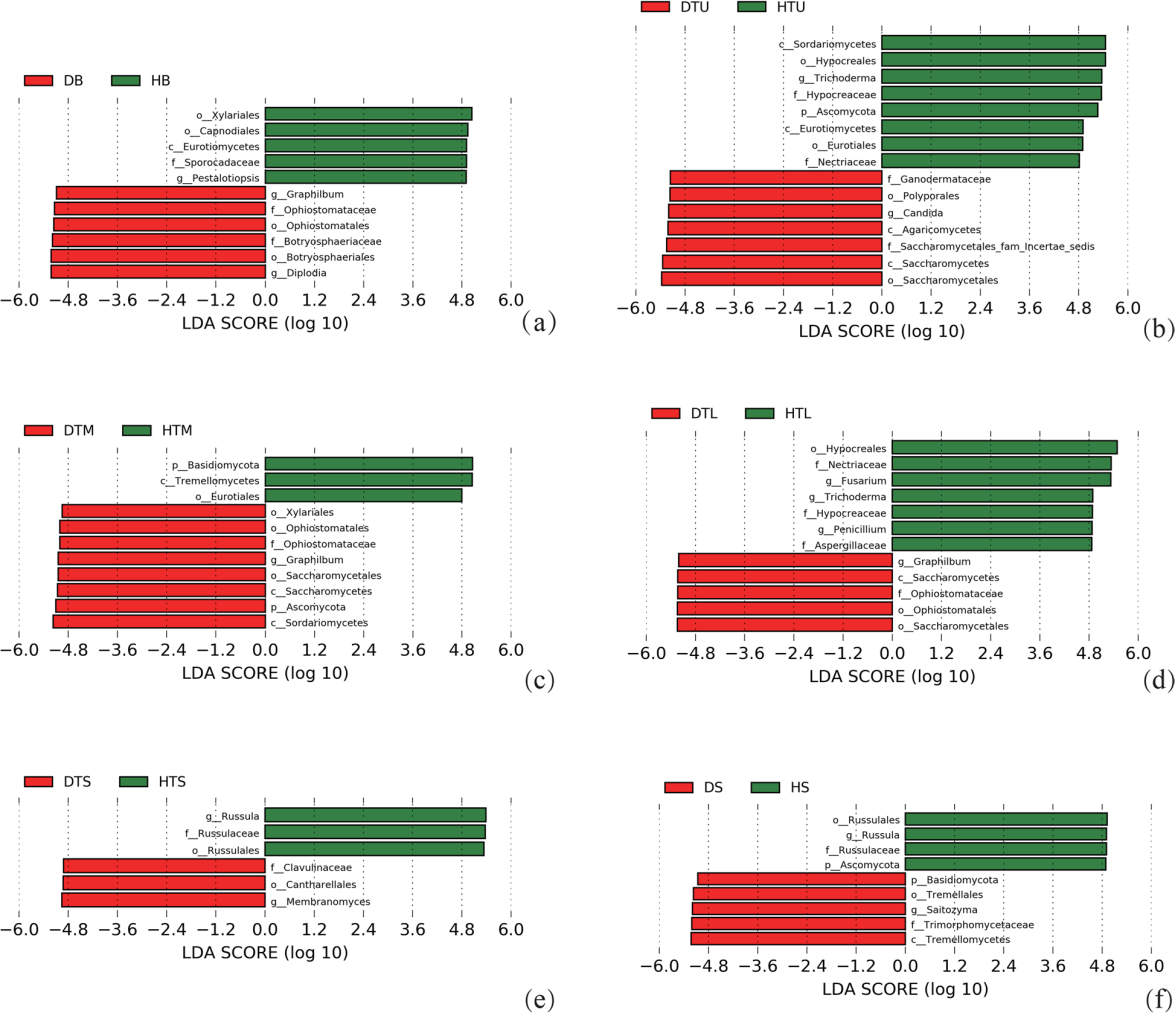
LEfSe analysis reveals substantial differences between diseased and healthy trees at fungal taxonomic levels in the branches (HB or DB), upper trunks (HTU or DTU), middle trunks (HTM or DTM), lower trunks (HTL or DTL), surface soil (HTS or DTS), and deep soil (HTS or DTS) (HS or DS). Abbreviation: p: phylum, c: class, f: family, o: order, and g: genus

The LEfSe analysis showed that the abundance of some fungal taxa differed between the healthy and diseased samples in the branches (HB or DB), upper trunks (HTU or DTU), middle trunks (HTM or DTM), lower trunks (HTL or DTL), surface soil (HTS or DTS) and deep soil (HS or DS), respectively (LDA > 4.8, *p* < 0.05) (Fig. 4). In the branches, the class Eurotiomycetes, the orders Capnodiales and Xylariales, the family Sporocadaceae, and the genus *Pestalotiopsis* were more abundant in the healthy trees, whereas the orders Ophiostomatales and Botryosphaeriaceae; the families Ophiostomataceae and Botryosphaeriaceae, the genera *Graphilbum* and *Diplodia* were had a higher abundance in the diseased trees (Fig. 4a). In the upper trunks, the phylum Ascomycota, the classes Sordariomycetes and Eurotiomycetes, the orders Hypocreales and Eurotiales, the families Hypocreaceae and Nectriaceae, and the genus *Trichoderma* were more abundant in the healthy trees, whereas the classes Saccharomycetes and Agaricomycetes, the orders Saccharomycetales and Polyporales, the family Ganodermataceae, and the genus *Candida* were had a higher abundance in the diseased trees (Fig. 4b). In the middle trunks, the phylum Basidiomycota, the class Tremellomycetes, and the order Eurotiales were more abundant in the healthy trees, whereas the phylum Ascomycota, the classes Sordariomycetes and Saccharomycetes, the orders Saccharomycetales, Ophiostomatales and Xylariales; the family Ophiostomataceae, and the genus *Graphilbum* were had a higher abundance in the diseased trees (Fig. 4c). In the lower trunks, the order Hypocreales, the families Nectriaceae, Hypocreaceae and Aspergillaceae; and the genera *Fusarium*, *Trichoderma* and *Penicillium* were more abundant in the healthy trees, whereas the class Saccharomycetes, the orders Saccharomycetales and Ophiostomatales; the family Ophiostomataceae, and the genus *Graphilbum* were had a higher abundance in the diseased trees (Fig. 4d). In the surface soil, the order Russulales, the family Russulaceae, and the genus *Russula* were more abundant in the healthy trees, whereas the order Cantharellales, the family Clavicipitaceae, and the genus *Membranomyces* were had a higher abundance in the diseased trees (Fig. 4e). In the deep soil, the phylum Ascomycota, the order Russulales, the family Russulaceae, and the genus *Russula* were more abundant in the healthy trees, whereas the phylum Basidiomycota, the class Tremellomycetes, the order Tremellales, the family Trimorphomycetaceae, and the genus *Saitozyma* were had a higher abundance in the diseased trees (Fig. 4f).

Analysis of the abundance of fungal taxa in different parts of healthy trees are as follows. The class Dothideomycetes, the orders Xylariales, Botryosphaeriaceae and Chaetothyriales; the families Sporocadaceae, Teratosphaeriaceae, Botryosphaeriaceae, Cladosporium and Mycosphaerellaceae; the genera *Pestalotiopsis*, *Devriesia*, *Diplodia* and *Cladosporium* were more abundant in the branches. The family Hypocreaceae and the genus *Trichoderma* were more abundant in the upper trunks. The class Saccharomycetes, the orders Saccharomycetales, Pleosporales and Cantharellales; and the genus *Candida* were more abundant in the middle trunks. The phylum Ascomycota, the class Sordariomycetes, the order Hypocreales, the family Nectriaceae, and the genus *Fusarium* were more abundant in the lower trunks. The phyla Basidiomycota, the class Agaricomycetes, the order Atheliales, the families Clavicipitaceae, Atheliaceae and Herpotrichiellaceae; the genera *Cladophialophora* and *Tylospora* were more abundant in the surface soil. The phyla Mortierellomycota, the classes Mortierellomyceres and Leotiomycetes; the orders Tremellales, Mortierellales and Helotiales; the families Trimorphomycetaceae and Mortierellaceae; the genera *Saitozyma* and *Mortierella* were more abundant in the deep soil (Fig. S1a).

Analysis of the abundance of fungal taxa in different parts of disease trees are as follows. The order Botryosphaeriales, the families Botryosphaeriaceae, Bionecteiaceae and Chrysozymaceae; the genera *Diplodia*, *Capronia*, *Ophiostoma*, *Hamamotoa* and *Clonostachys* were more abundant in the branches. The classes Agaricomycetes and Saccharomycetes, the orders Saccharomycetales and Polyporales, the families Ganodermataceae and Pichiaceae, and the genus *Kuraishia* were more abundant in the upper trunks. The phyla Ascomycota, the class Sordariomycetes, the orders Hypocreales and Xylariales; the families Nectriaceae, Hypocreaceae and Sporocadaceae; the genera *Xenoacremonium*, *Trichoderma*, *Trigonosporomyces*, *Fusarium* and *Neopestalotiopsis* were more abundant in the middle trunks. The orders Ophiostomatales, Russulales and Eurotiales; the families Ophiostomataceae and Trichocomaceae, and the genus *Talaromyces* were more abundant in the lower trunks. The phyla Basidiomycota and Mortierellomycota; the classes Mortierellomycetes and Cystobasidiomycetes; the orders Mortierellales, Chaetothyriales and Filobasidiales; the families Mortierellaceae, Aspergillaceae, Russulaceae, Clavicipitaceae, Herpotrichiellaceae and Teratosphaeriaceae; the genera *Penicillium*, *Mortierella*, *Pestalotiopsis* and *Devriesia* were more abundant in the surface soil. The classes Tremellomycetes and Leotiomycetes; the orders Tremellales, Helotiales and Trichosporonales; the families Trimorphomycetaceae and Trichosporonaceae; the genera *Saitozyma* and *Apiotrichum* were more abundant in the deep soil (Fig. S1b).

**Fig. 5:**
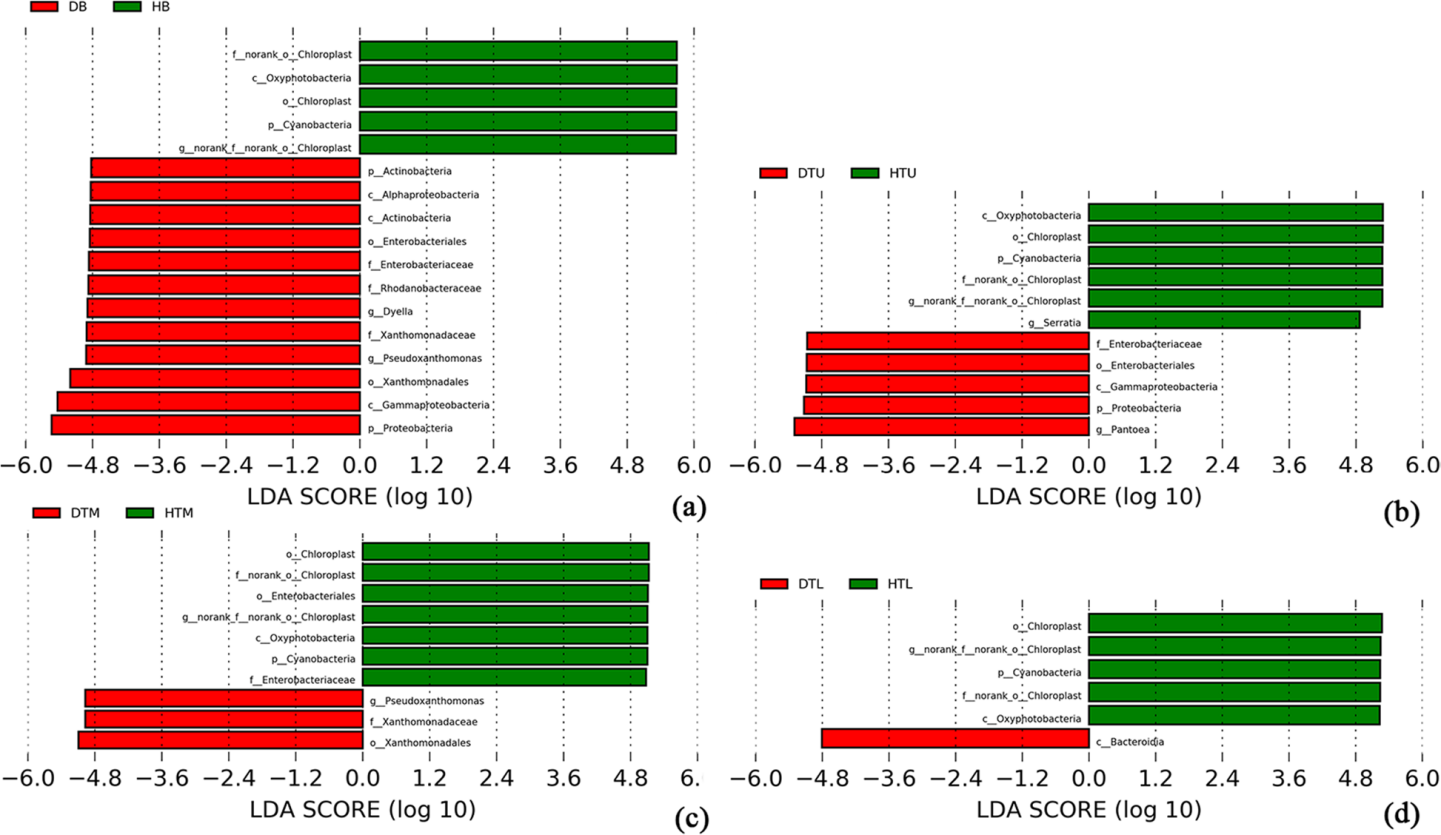
LEfSe analysis showing the significant differences at bacterial taxonomic levels between diseased and healthy trees in the branches (HB or DB), upper trunks (HTU or DTU), middle trunks (HTM or DTM), and lower trunks (HTL or DTL). Abbreviation: p: phylum, c: class, f: family, o: order, and g: genus

The LEfSe analysis showed that the abundance of some bacterial taxa differed between the healthy and diseased samples in the branches (HB or DB), upper trunks (HTU or DTU), middle trunks (HTM or DTM), lower trunks (HTL or DTL), surface soil (HTS or DTS) and deep soil (HS or DS), respectively (LDA > 4.8, *p* < 0.05) (Fig. 5). In the branches, the phylum Cyanobacteria, the class Oxyphotobacteria and the order Chloroplast were more abundant in the healthy tree, whereas the phyla Proteobacteria and Actinobacteria, the classes Gammaproteobacteria, Actinobacteria and Alphaproteobacteria; the orders Xanthomonadales and Enterobacteriales; the families Xanthomonadaceae, Rhodanobacteraceae and Enterobacteriaceae; the genus *Pseudoxanthomonas* and *Dyella* were had a higher abundance in the diseased tree (Fig. 5a). In the upper trunks, the phylum Cyanobacteria, the class Oxyphotobacteria, the order Chloroplast, and the genera *Serratia* were more abundant in the healthy trees, whereas the phylum Proteobacteria, the class Gammaproteobacteria, the order Enterobacteriales, the family Enterobacteriaceae, and the genera *Pantoea* were had a higher abundance in the diseased trees (Fig. 5b). In the middle trunks, the phylum Cyanobacteria, the class Oxyphotobacteria, the orders Chloroplast and Enterobacteriales, and the family Enterobacteriaceae were more abundant in the healthy trees, whereas the order Xanthomonadales, the family Xanthomonadaceae, and the genera *Pseudoxanthomonas* were had a higher abundance in the diseased trees (Fig. 5c). In the lower trunks, the phylum Cyanobacteria, the class Oxyphotobacteria, and the order Chloroplastwere more abundant in the healthy trees, whereas the class Bacteroidia was had a higher abundance in the diseased trees (Fig. 5d).

Analysis of the abundance of bacterial taxa in different parts of the healthy trees are as follows. The phylum Cyanobacteria, the class Oxyphotobacteria, the order Chloroplast were more abundant in the branches. The genus *Serratia* was more abundant in the upper trunks. The phylum Proteobacteria, the class Gammaproteobacteria, the order Enterobacteriales and the family Enterobacteriaceae were more abundant in the middle trunks. The order Betaproteobacteriales, the family Burkholderiaceae, and the genus *Burkholderia-Caballeronia-Paraburkholderia* were more abundant in the lower trunks. The phyla Actinobacteria, the classes Alphaproteobacteria and Actinobacteria were more abundant in the surface soil. The phyla Acidobacteria and the class Acidobacteriia were more abundant in the deep soil (Fig. S1c). Analysis of the abundance of bacterial taxa in different parts of the disease trees. The order Xanthomonadales, the family Rhodanobacteraceae, and the genus *Dyella* were more abundant in the branches. The phyla Proteobacteria, the class Gammaproteobacteria, the order Enterobacteriales, the family Enterobacteriaceae, and the genus *Pantoea* were more abundant in the upper trunks. The family Xanthomonadaceae and the genus *Pseudoxanthomonas* were more abundant in the middle trunks. The orders Ophiostomatales, Russulales and Eurotiales; the families Ophiostomataceae and Trichocomaceae, and the genus *Talaromyces* were more abundant in the lower trunks. The class Alphaproteobacteria was more abundant in the surface soil. The phyla Actinobacteria and the class Actinobacteria were more abundant in the deep soil (Fig. S1b).

### Microbial correlation of diseased and healthy trees

**Fig. 6:**
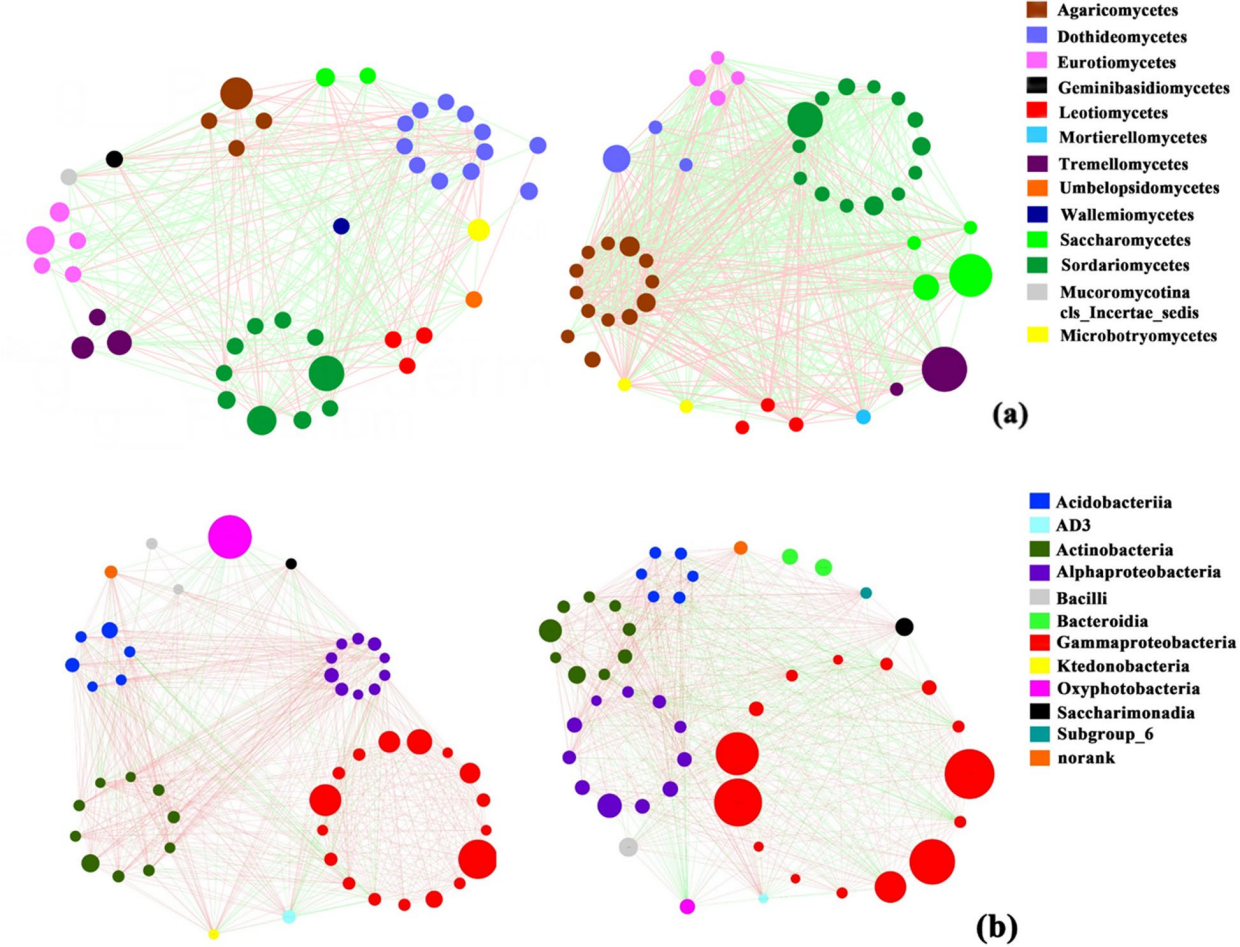
In *P. massoniana*, there is a correlation displaying the relative abundance of the microbial community. **a** shows the relationship between fungus in healthy and damaged plants. **b** show the relationship between bacteria in healthy and diseased trees. The color symbolizes classes, whereas the nodes represent genera. The positive correlation is shown by the red lines, while the negative correlation is represented by the green lines. The absolute value of correlation > 0.5 and *p*-value< 0.05

Fungal correlation results showed there were 334 strong taxon–taxon correlations in healthy trees, positive correlation demonstrated a double (218 vs. 116, ratio = 1.88) increase in the number of negative correlations among them. There were 707 strong taxon–taxon correlations in diseased trees, positive correlation demonstrated an equal (406 vs. 301, ratio = 1.35) number of negative correlations among them. At the phylum level, Ascomycota has the most correlations degrees (cd) (778) in both type of trees. At the class level, Microbotryomycetes had significant correlation only in healthy trees, however Geminibasidiomycetes, Umbelopsidomycetes and Wallemiomycetes were closed in diseased trees. Interestingly, although both groups contain Agaricomycetes and Sordariomycetes, the degrees in healthy trees were an obviously increase in diseased trees, they are 381:87 and 314:118 respectively (Fig. 6a). Among the diseased trees, some genera with the highest abundance were *Membranomyces*, *Oidiodendron* and *Ganoderma*. Also, some genera such as *Membranomyces*, *Ganoderma*, *Tomentella*, *Menispora*, *Ophiostoma*, *Hamamotoa*, *Graphilbum*, *Xenoacremonium*, *Cytospora*, *Clonostachys* and *Entomocorticium* were only exist in diseased trees. Among the healthy trees, some genera with the highest abundance were *Candida* (27), *Geminibasidium* (23) and *Mortierella* (23). Also, there were some genera such as *Geminibasidium*, *Bifiguratus*, *Fusarium*, *Cladosporium*, *Lasiodiplodia*, *Tylospora*, *Phialemoniopsis*, *Umbelopsis*, *Paraconiothyrium*, *Capnobotryella*, *Neopestalotiopsis*, *Pestalotiopsis*, *Catenulostroma* and *Wallemia*. Surprisingly, although *Apiotrichum*, *Devriesia*, *Kuraishia*, *Penicillium* and *Trichoderma* have existed both diseased and healthy trees, the abundance of *Apiotrichum*, *Devriesia*, *Kuraishia* and *Penicillium* in diseased trees were triple to fivefold as in healthy trees, the abundance of *Trichoderma* in healthy trees were triple as in diseased trees.

Bacterial correlation results showed there were 637 strong taxon–taxon correlations in healthy trees, positive correlation demonstrated a fivefold (535 vs. 102, ratio = 5.25) increase in the number of negative correlations (absolute value of Spearman correlation > 0.5 and false discovery rate-corrected *p* < 0.05) among them. There were 866 strong taxon–taxon correlations in diseased trees, positive correlation demonstrated equal (465 vs. 401, ratio = 1.01) the number of negative correlation (absolute value of Spearman correlation > 0.5 and false discovery rate-corrected p < 0.05) among them. At the phylum level, Proteobacteria has the most degrees in both healthy (641) and diseased trees (805). At the class level, Ktedonobacteria exists in healthy trees, while Bacteroidia exists only in diseased trees. Interestingly, Oxyphotobacteria only owned one node in the figure, but the size in healthy trees was tenfold (30 vs. 3, ratio = 10) bigger than it in diseased trees (Fig. 6b). The bacteria group displayed a co-occurrence network with a strong positive correlation among genera. Among the diseased trees, some genera with the highest abundance were *Acidothermus*, *Enterobacter* and *Pseudomonas*. Also, some genera such as *Erwinia*, *Fluviicola*, *Novosphingobium*, *Gryllotalpicola*, *Nocardioides*, *Terriglobus*, *Acidipila*, *Curtobacterium*, *Chitinophaga*, *Lactobacillus* and *Edaphobacter* were only exist in diseased trees. Among the healthy trees, some genera with the highest abundance were *Candidatus* (34), *Ralstonia* (34) and *Rhodococcus* (34). Also, some genera such as *Rhodococcus*, *Occallatibacter*, *Kosakonia*, *Brevundimonas*, *Massilia*, *Silvimonas*, *Serratia* and *Stenotrophomonas* were only exist in diseased trees. Surprisingly, although *Dyella* and *Pantoea* have existed between diseased and healthy trees, the abundance in diseased trees was twice as in healthy trees.

**Fig. 7:**
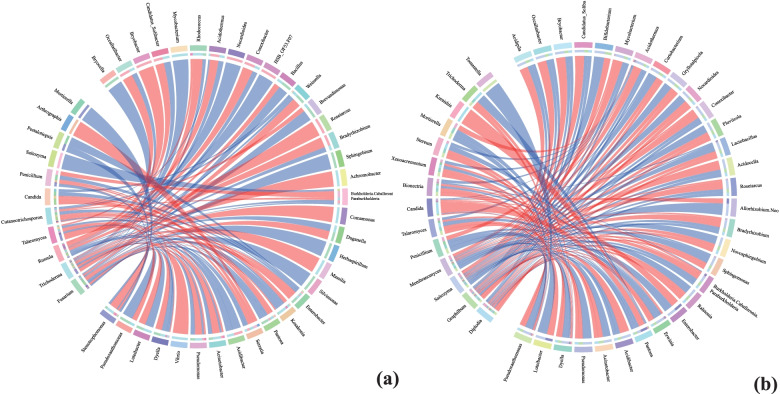
In healthy and diseased trees, the microbiota differs. **a** projected the relationship between fungi and bacteria in healthy trees, and (**b**) projected the relationship between fungi and bacteria in diseased trees. The color symbolizes classes, whereas the nodes represent genera. The positive correlation is shown by the orange lines, while the negative correlation is represented by the blue lines

We constructed the correlation model of bacteria and fungi in healthy trees and diseased trees by SPLS. The results showed in healthy trees, *Trichoderma* and *Stenotrophomonas* are positively correlated and have the highest correlation (the SPLS coefficient is 4.08), and *Fusarium* and *Pantoea* are negatively correlated and have the highest correlation (the SPLS coefficient is − 1.96). However, In the disease trees, *Candida* and *Pantoea* are positively correlated and have the highest correlation (the SPLS coefficient is 0.75), and *Saitozyma* and *Pseudoxanthomonas* are negatively correlated and have the highest correlation (the SPLS coefficient is − 0.78). In addition, we found that bacteria with high abundance in healthy trees as *Kosakonia*, *Brevundimonas* and *Serratia* were positively correlated with fungi *Cutaneotrichosporon* (the SPLS coefficients are 0.38, 0.33 and 0.27), while they were negatively related with fungi *Russula* (the SPLS coefficients are − 0.14, − 0.19 and − 0.23) (Fig. 7a). Bacteria that were more abundant in diseased trees as *Erwinia*, *Fluviicola*, *Novosphingobium*, *Gryllotalpicola*, *Nocardioides*, *Lactobacillus* and *Dyella* were positively correlated with fungi *Graphilbum* (the SPLS coefficients are 0.21, 0.04, 0.05, 0.03, 0.05, 0.14 and 0.39), while they were negatively related with fungi *Saitozyma* (the SPLS coefficients are − 0.47, − 0.06, − 0.10, − 0.06, − 0.08, − 0.22 and − 0.71) (Fig. 7b). They are important candidate microorganisms involved in the pathological mechanism of PWN.

### Microbial function of healthy and diseased trees

**Fig. 8:**
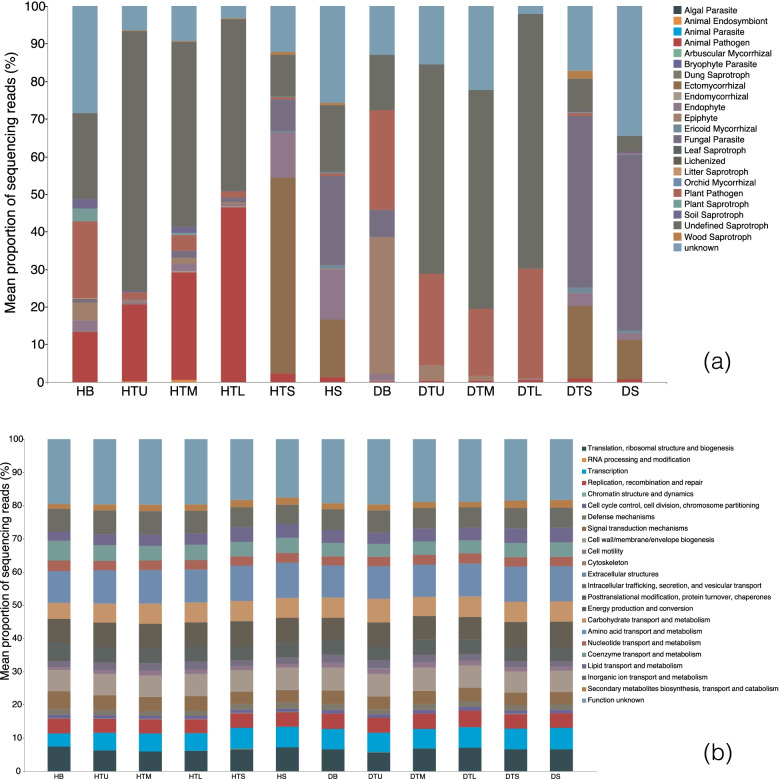
FUNGuild and COG for the predicted resource utilization function. Branches are represented by HB and DB, upper trunk by HTU and DTU, middle trunk by HTM and DTM, lower trunk by HTL and DTL, surface soil by HTS and DTS, and deep soil by HS and DS

There were 649 OTUs (22.9%) matched in the FUNGuild analysis for the predicted resource utilization function of fungi. These OTUs were assigned to 20 functional guilds. Overall, the guild animal pathogen had the highest abundance (24.8%), followed by endophyte (16.5%), plant-pathogen (14.9%), ectomycorrhizal (13.7%), fungal parasite (9.2%), wood saprotroph (6.5%), ericoid mycorrhizal (3.1%), soil saprotroph (2.2%), dung saprotroph (1.7%), plant saprotroph (1.5%), epiphyte (1.5%), animal endosymbiont (1.5%), arbuscular mycorrhizal (0.9%), orchid mycorrhizal (0.5%), leaf saprotroph (0.5%), bryophyte parasite (0.3%), litter saprotroph (0.2%), lichenized (0.2%), animal parasite (0.2%) and algal parasite (0.2%) (Fig. 8a). In healthy trees, animal pathogen, plant pathogen and saprotroph are the top three functional guilds. Among them, animal pathogen (46.39%) is mainly concentrated in the lower trunk, plant pathogen (20.39%) is mainly concentrated in the branches, and saprotroph (69.82%) is mainly concentrated in the upper trunk. However, plant pathogen, parasite and saprotroph are the top three functional guilds of diseased trees. Among them, plant pathogen (29.12%) is mainly concentrated in the lower trunk, parasite (36.52%) is mainly concentrated in the branches, and saprotroph (67.88%) is mainly concentrated in the upper trunk. Interestingly, parasitic fungus in soil of diseased trees (16.43%) is significantly higher than soil of healthy trees (6.49%), and the endophyte in soil of diseased trees (5.08%) is significantly less than soil of healthy trees (0.92%).

The COG analysis was utilized for the predication of resource utilization function of bacteria. The functions of COG were assigned to 23 functional guilds. Among them, the guild amino acid transport and metabolism had the highest abundance (10.1%), followed by energy production and conversion (7.3%), cell wall/membrane/envelope biogenesis (6.6%), translation, ribosomal structure and biogenesis (6.6%), inorganic ion transport and metabolism (6.4%), carbohydrate transport and metabolism (6.0%), transcription (5.7%), replication, recombination and repair (4.5%), posttranslational modification, protein turnover, chaperones (4.5%), coenzyme transport and metabolism (4.4%), signal transduction mechanisms (4.1%), lipid transport and metabolism (3.7%), nucleotide transport and metabolism (2.9%), intracellular trafficking, secretion, and vesicular transport (2.1%), secondary metabolites biosynthesis, transport and catabolism (1.9%), defense mechanisms (1.7%), cell motility (1.3%), cell cycle control, cell division, chromosome partitioning (1.0%), and others (< 1.0%) (Fig. 8b).

**Fig. 9:**
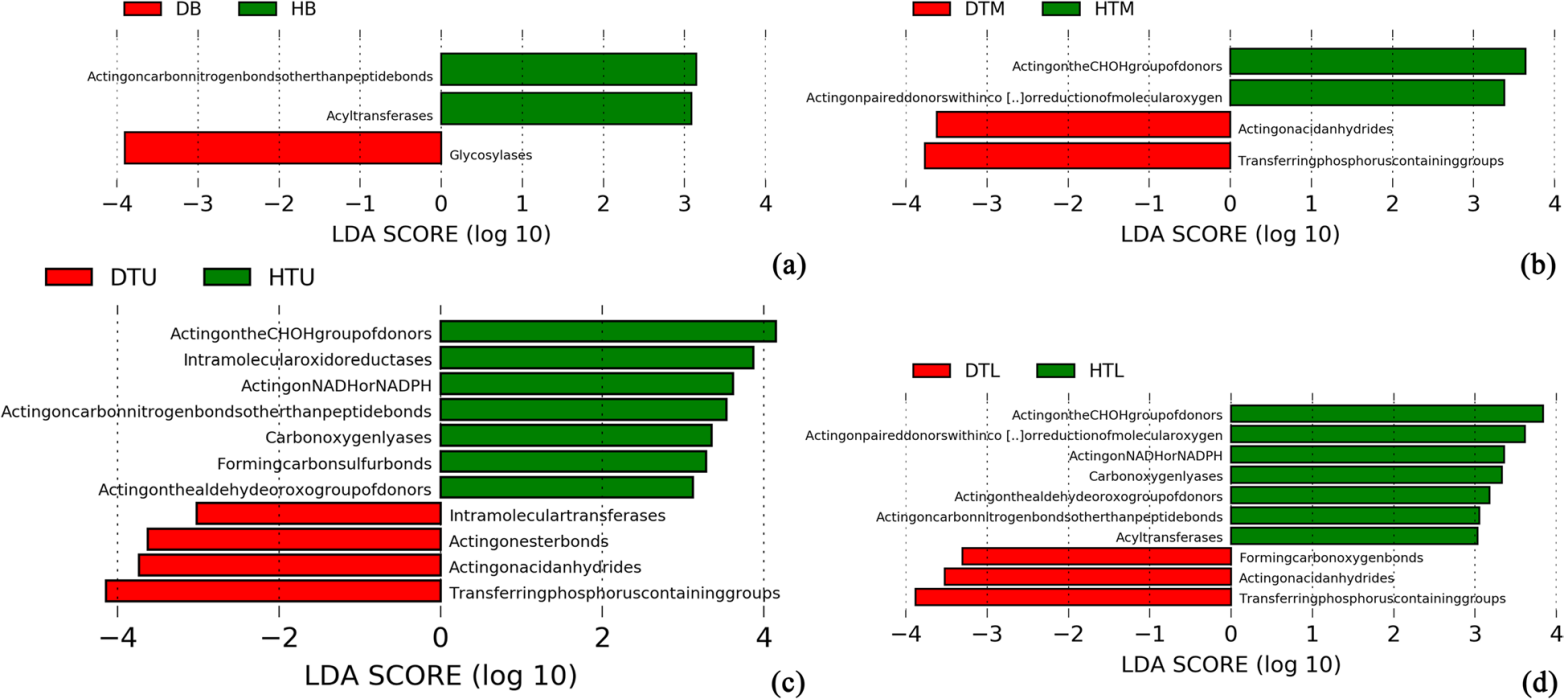
LEfSe analysis showed the predicted functional groups significantly presented for healthy and diseased samples in the branches (**a**), upper trunks (**b**), middle trunks (**c**) and lower trunks (**d**). Branches are represented by HB and DB, upper trunk by HTU and DTU, middle trunk by HTM and DTM, lower trunk by HTL and DTL

The results of PICRUSt2 analysis for the predicted function showed the OTUs were assigned to six functional groups and 52 sub-groups. The top functional annotations among them were the group Hydrolases (34.72%), followed by transferases (23.35%), oxidoreductases (23.34%), isomerases (6.94%), ligases (6.03%) and lyases (5.61%). The LEfSe analysis showed that the abundance of some functional groups differed in abundance between the diseased and healthy samples in the branches, trunks, and soil, respectively (LDA > 3.0, *p* < 0.05). In the branches, the abundance of Acting on carbon-nitrogen bonds other than peptide bonds and the acyltransferases were higher in healthy branches, whereas the glycosyltransferases had a higher abundance in diseased branches (Fig. 9a). In the upper trunks, the abundance of acting on the CH-OH group of donors, intramolecular oxidoreductases, acting on NADH or NADPH, acting on carbon-nitrogen bonds other than peptide bonds, carbon-oxygen lyases, forming carbon-sulfur bonds and acting on the aldehyde or oxo group of donors was higher in healthy upper trunks, whereas the intramolecular transferases, acting on ester bonds, acting on acid anhydrides and transferring phosphorus-containing groups had a higher abundance in diseased upper trunks (Fig. 9b). In the middle trunks, the abundance of acting on the CH-OH group of donors and acting on paired donors with incorporation or reduction of molecular oxygen was higher in healthy middle trunks, whereas the acting on acid anhydrides and transferring phosphorus-containing groups had a higher abundance in diseased middle trunks (Fig. 9c). In the lower trunks, acting on the CH-OH group of donors, acting on paired donors with incorporation or reduction of molecular oxygen, acting on NADH or NADPH, carbon-oxygen lyases, acting on the aldehyde or oxo group of donors and acting on carbon nitrogen bonds other than peptide bonds were higher in healthy trunks, whereas the forming carbon-oxygen bonds, acting on acid anhydrides and transferring phosphorus-containing groups had a higher abundance in diseased trunks (Fig. 9d).

## Discussion

Microorganisms play an important role in plants, since combined action of endophytes and pathogens work together to cause changes in plant tissues [39, 40]. Invasion by external items (such as insects) can alter the structure of the microbial community that lives on plants. In this article, the microbial community composition, organization, correlation, and function of healthy pines and pines infected by PWN in natural settings were all described. Combined with previous research, it was shown that PWN infection had a significant influence on the microbial community of the host plant, as well as the microbial structure of the host plant.

We discovered various changes in branches, trunks, and soil between healthy and diseased trees in the current study. The richness, evenness, and diversity of the microbial community in diseased trees were higher than in healthy trees, according to our findings, which are comparable to those on leaves [20]. We also looked at the microbial community composition at the phylum and class levels, and discovered that PWN infection might drastically alter the number of microbial communities in branches and trunks rather than soil. Microbial diversity in the pine micro ecological environment has been linked to the distinct periods when PWN infected the host plant in previous research [41]. There was no significant change in bacterial diversity between healthy trees and early diseased trees in the research of *P. thunbergii*, *P. massoniana* and *P. koraiensis* [16, 18, 42]. Most crucially, time has a higher impact on the makeup of the microbial community than the developmental stage of the host [43]. The diseased tree samples gathered in this study, on the other hand, are thought to be in the middle or late stages of PWN infection.

The unevenness of particular taxa in distinct microbial communities influenced the organisms’ microbial ecology [44]. We discovered that the principal fungal genera (*Trichoderma*, *Fusarium*, *Russula* and *Penicillium*) and bacterial genera (*Serratia* and *Burkholderiaceae*) in healthy trees were located in the microbial community structure of branches, trunks, and soil. These genera are crucial in the biological control of nematodes, because they may create traps or metabolites that are detrimental to nematodes [45–47]. Plant pathogenic microorganisms include the primary fungal genera (*Saitozyma*, *Graphilbum*, *Diplodia* and *Candida*) and bacterial genera (*Pseudoxanthomonas, Dyella* and *Pantoea*) found in diseased plants. Based on these results and previous studies of others, we suggested that PWN reduced the pines’ defensive capabilities by altering the endophytic composition. However, the possibility of PWN directly reduced pines’ defensive capabilities and then accelerate the alter of endophytic composition, and then work with the community to hasten the demise of the pines do exist. Constructing artificial communities under laboratory conditions may be a practical way to do get insights into these possibilities, which is worth further study in the future.

Through the correlation prediction, we found that PWN infection has a great impact between fungi and bacteria. Fungal genera such as *Membranomyces*, *Ganoderma*, *Tomentella*, *Menispora*, *Ophiostoma*, *Hamamotoa*, *Graphilbum*, *Ogataea*, *Geosmithia*, *Xenoacremonium*, *Cytospora*, *Clonostachys*, *Entomocorticium* were highly abundant only in the diseased trees. Among them, *Ophiostoma*, *Graphilbum*, *Cytospora* and *Clonostachys* are associated with pine infected by *B. xylophilus* [48–50]. Meanwhile, fungal genera such as *Geminibasidium*, *Bifiguratus*, *Fusarium*, *Cladosporium*, *Lasiodiplodia*, *Tylospora*, *Phialemoniopsis*, *Umbelopsis*, *Paraconiothyrium*, *Capnobotryella*, *Neopestalotiopsis*, *Pestalotiopsis*, *Catenulostroma*, *Wallemia* were highly abundant only in the healthy trees. Among them, *Geminibasidium*, *Cladosporium*, *Phialemoniopsis*, *Umbelopsis*, *Paraconiothyrium* can promote plant growth and nutrient accumulation [51–55]. In this study, although bacteria genera *Acidothermus*, *Enterobacter* and *Pseudomonas* with the higher abundance in diseased trees; bacteria genera *Candidatus*, *Ralstonia* and *Rhodococcus* with the higher abundance in healthy trees; those genera are all common bacterial genera in the environment. We found *Erwinia*, *Fluviicola*, *Novosphingobium*, *Gryllotalpicola*, *Nocardioides*, *Terriglobus*, *Acidipila*, *Curtobacterium*, *Chitinophaga* in diseased trees. *Lactobacillus*, *Edaphobacter* and other genera are more abundant than those found in healthy trees. It is interesting to note that extracts of *Erwinia*, *Novosphingobium* and *Gryllotalpicola* can effectively kill nematodes [56, 57]. These bacterial genera may be a potential alternative to PWD by inducing systemic resistance in pines. Meanwhile, the abundance of *FCPS473*, *Rhodococcus*, *Occallatibacter*, *Kosakonia*, *Brevundimonas*, *Burkholderia*, *Massilia*, *Silvimonas*, *Serratia*, *Stenotrophomonas*, *Herbaspirillum*, *Streptomyces*, *Duganella*, *Bacillus* and other bacteria in healthy trees was higher than that in diseased trees. Among them, *Rhodococcus*, *Kosakonia*, *Massilia*, *Stenotrophomonas* and *Bacillus* are plant probiotics [58–61]. These endophytic bacteria affect improving plant adaptability and PWD tolerance.

Invasion of PWN has a stronger influence on fungi than bacteria, according to the function study [62]. After the invasion of PWN, the metabolic function of the fungal population in trunks altered. In diseased trees, the function of DNA-directed DNA & RNA polymerase, as well as its ability to act on acid anhydrides to limit transmembrane transport of chemicals, altered dramatically. We hypothesized that PWN infection influenced resin secretion in pine trunks, resulting in a failure to close wounds in a timely manner, impede microorganism development, and lower the palatability of wood-boring insects. As a result, the likelihood of insects carrying PWN invaded trees has increased significantly, the tree potential has decreased, and the withering and mortality of pines will be exacerbated. These findings confirm the theory that microorganisms play a substantial role in the incidence of PWD when PWN infects pines. This set of observations lends credence to the idea that enzyme theory and toxin theory have a role in the pathophysiology of PWN. As a result, follow-up research with a stronger focus on PWN is recommended.

Finally, the species and population of microbial communities in the branches and trunks of *P. massoniana*, but not in the soil, altered dramatically. This might be linked to infection mode of PWN. Interestingly, microorganisms such as *Trichoderma*, *Fusarium*, *Russula*, *Penicillium*, *Serratia* and *Burkholderiaceae* were mainly found in healthy trees, and the pathogens such as *Saitozyma*, *Graphilbum*, *Diplodia*, *Candida*, *Pseudoxanthomonas*, *Dyella* and *Pantoea* were mainly found in diseased trees. We speculate that the infection of PWN destroys the microbial defense barrier of pines. It improved the infection ability of pathogenic microorganisms to pines and accelerated the death of pines. Spearman’s correlation analysis revealed that the infection of PWN changed *Apiotrichum*, *Devriesia*, *Kuraishia*, *Penicillium*, *Trichoderma, Dyella and Pantoea* in pine. PWN greatly changed the microbial environment of *P. massoniana* and disrupted the homeostasis of pines. Turpentine is high in anhydrides and acid, both of which are beneficial for the tree’s disease resistance. We discovered that the microbial community increased the degradation of turpentine in diseased trees as a functional prediction. The rise in glycosyltransferases also revealed that the microbial community aided the PWN in speeding up the pine invasion. Following that, we hypothesized that PWN infiltrated pine and disrupted the composition and organization of the endophytic microbial community, resulting in a drop in probiotics and an increase in pathogenic bacteria, based on the results of functional analysis. The coordinated development of pine wilt disease following PWN invasion is elucidated at the microbiological level in this work. This study adds to our understanding of the etiology of pine wilt illness. More information about PWN would aid us in achieving a higher level of accuracy in this regard.

## Supplementary Information

**Additional file 1.**

## Data Availability

All analyses tools used in the study are publicly available. The datasets generated and analysed during the current study are available in the Sequence Read Archive (SRA) repository at the National Center for Biotechnology Information (https://submit.ncbi.nlm.nih.gov/subs/sra/), with the accession number PRJNA 768116.

## Declarations

All methods were performed in accordance with the relevant guidelines and regulations.
