# Supplementary material for: Effects on community composition and function Pinus massoniana infected by Bursaphelenchus xylophilus
Source: BMC Microbiol. 2022 Jun 11;22:157. doi: 10.1186/s12866-022-02569-z (PMC9188149; doi:10.1186/s12866-022-02569-z)
Supplement: Supplementary file 1 — Additional file 1. [file 12866_2022_2569_MOESM1_ESM.docx]

Supplementary Material

# Supplementary Figures


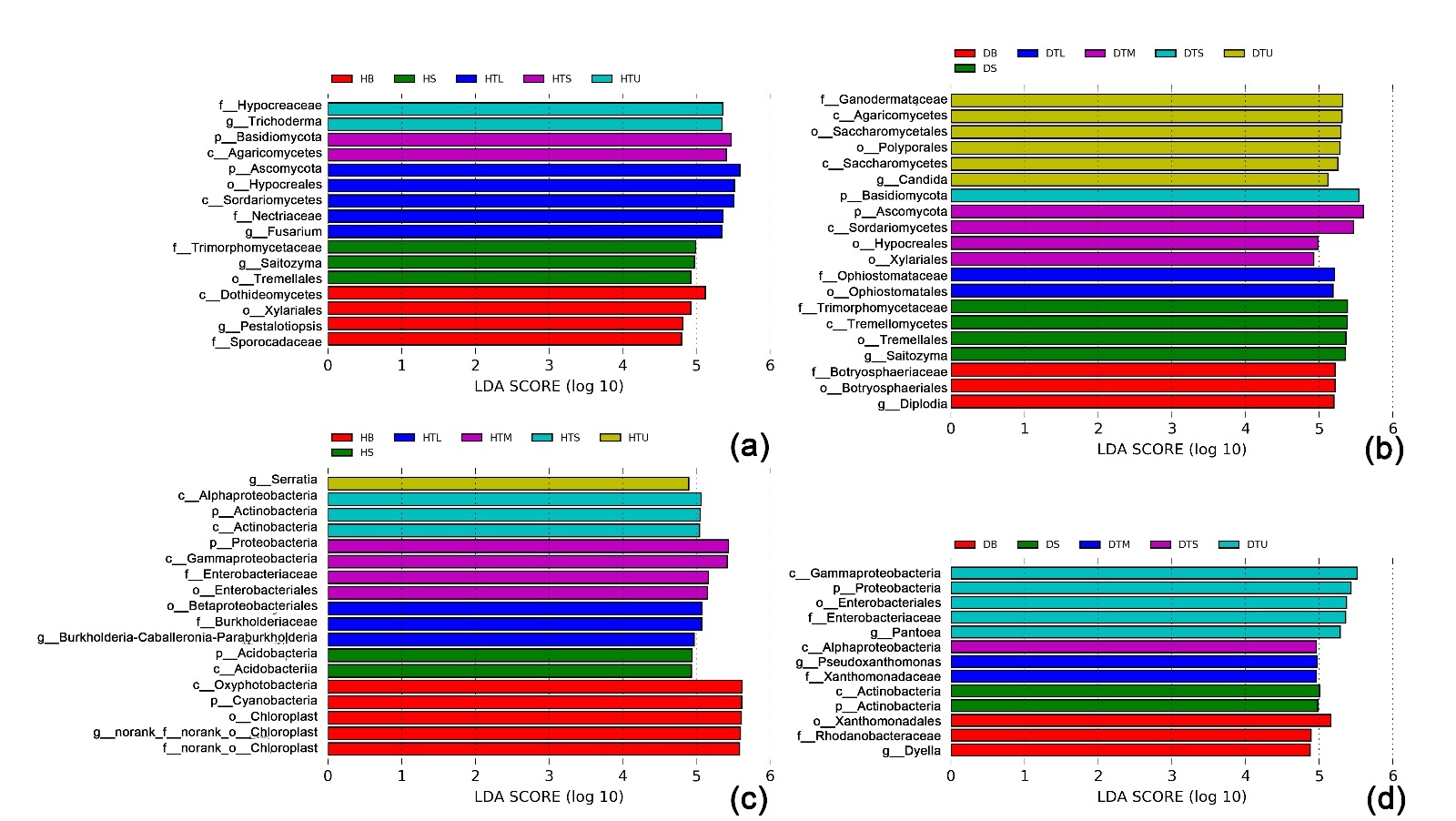


**Figure S1.** Abundance of some taxa in different parts of the healthy and disease trees. (a) Abundance of fungi in different parts of the healthy trees. (b) Abundance of fungi in different parts of the disease trees. (c) Abundance of bacteria in different parts of the healthy trees. (d) Abundance of bacteria in different parts of the disease trees. Abbreviation: HB and DB represent branches, HTU and DTU represent upper trunk, HTM and DTM represent middle trunk, HTL and DTL represent lower trunk, HTS and DTS represent surface soil, and HS and DS represent deep soil. p: phylum, c: class, f: family, o: order, and g: genus.

# Supplementary Tables

**Table S1.** The numbers of microbial community between diseased (D) and healthy (H) trees

| Microorganism | Samples | Phylum | Class | Order | Family | Genus | Species | OTUs |
| --- | --- | --- | --- | --- | --- | --- | --- | --- |
| Fungi | Diseased | 10 | 35 | 86 | 197 | 338 | 491 | 1837 |
|  | Healthy | 14 | 44 | 105 | 215 | 369 | 546 | 2150 |
| Bacteria | Diseased | 47 | 107 | 288 | 572 | 1173 | 2313 | 5762 |
|  | Healthy | 39 | 92 | 258 | 461 | 940 | 1758 | 4378 |

**Table S2.** The microbial community diversity estimators in the between diseased trees and healthy trees

| Samples | | Richness | Chao1 | Shannon | Shannon even |
| --- | --- | --- | --- | --- | --- |
| Diseased Tree  Fungi | Branches | 107±31 | 107.78±30.35 | 3.32±0.50 | 0.71±0.08 |
|  | Upper Trunks | 106±37 | 110.83±39.30 | 2.72±0.50 | 0.58±0.06 |
|  | Middle Trunks | 110±47 | 111.62±45.58 | 3.28±0.30 | 0.72±0.12 |
|  | Lower Trunks | 121±3 | 124.08±2.76 | 2.75±0.34 | 0.56±0.07 |
|  | Surface Soil | 765±103 | 918.99±70.43 | 3.06±0.65 | 0.45±0.09 |
|  | Deep Soil | 799±56 | 883.74±76.79 | 3.81±0.12 | 0.57±0.02 |
| Healthy Tree  Fungi | Branches | 106±8 | 155.59±48.05 | 1.92±0.03 | 0.39±0.01 |
|  | Upper Trunks | 90±18 | 106.66±26.77 | 1.51±0.56 | 0.32±0.10 |
|  | Middle Trunks | 92±5 | 102.41±9.78 | 2.05±0.05 | 0.44±0.02 |
|  | Lower Trunks | 106±1 | 110.52±3.17 | 1.96±0.22 | 0.41±0.04 |
|  | Surface Soil | 805±62 | 978.22±42.67 | 2.74±0.04 | 0.40±0.01 |
|  | Deep Soil | 519±96 | 654.10±100.85 | 2.49±0.65 | 0.39±0.09 |
|  | Branches | 611±70 | 677.73±91.29 | 3.94±0.29 | 0.62±0.06 |
|  | Upper Trunks | 527±99 | 723.69±158.81 | 3.17±0.11 | 0.51±0.01 |
| Diseased Tree | Middle Trunks | 793±368 | 852.68±403.74 | 4.55±0.63 | 0.69±0.08 |
| Bacteria | Lower Trunks | 1073±361 | 1130.44±367.35 | 4.56±0.70 | 0.65±0.07 |
|  | Surface Soil | 1999±108 | 2485.87±72.75 | 6.01±0.15 | 0.79±0.02 |
|  | Deep Soil | 1411±79 | 1730.76±115.93 | 5.54±0.16 | 0.76±0.02 |
|  | Branches | 320±83 | 392.88±57.32 | 0.86±0.78 | 0.15±0.03 |
|  | Upper Trunks | 434±128 | 476.49±113.08 | 2.89±0.20 | 0.48±0.02 |
| Healthy Tree | Middle Trunks | 559±127 | 633.21±161.90 | 3.45±0.41 | 0.55±0.08 |
| Bacteria | Lower Trunks | 444±146 | 463.21±152.29 | 3.01±0.67 | 0.49±0.09 |
|  | Surface Soil | 1850±117 | 2402.17±116.50 | 5.91±0.12 | 0.79±0.02 |
|  | Deep Soil | 1762±178 | 2173.97±171.36 | 5.91±0.09 | 0.79±0.00 |

All data are expressed as means ± standard deviation (n=3).

**Table S3.** Community composition at phylum level of fungi in diseased trees and healthy trees

| Phylum | Diseased Trees | | | | | |
| --- | --- | --- | --- | --- | --- | --- |
|  | DB | DTU | DTM | DTL | DTS | DS |
| Ascomycota | 87.53±1.66% | 54.30±28.96% | 99.51±0.19% | 88.05±3.02% | 18.59±38.26% | 13.75±7.23% |
| Basidiomycota | 0.75±0.45% | 33.43±36.54% | 0.39±0.17% | 10.52±3.14% | 69.74±31.00% | 68.99±6.03% |
| unclassified | 11.72±1.52% | 12.26±7.89% | 0.10±0.04% | 1.40±0.36% | 5.66±5.45% | 8.65±2.26% |
| Mortierellomycota |  | 0.01±0.01% |  | 0.03±0.04% | 3.26±1.34% | 1.44±1.05% |
| Rozellomycota |  |  |  |  | 2.39±2.59% | 7.08±2.28% |
| Mucoromycota |  |  |  |  | 0.36±0.19% | 0.08±0.07% |
| Phylum | Healthy Trees | | | | | |
|  | HB | HTU | HTM | HTL | HTS | HS |
| Ascomycota | 85.62±13.00% | 93.30±7.71% | 76.32±7.85% | 96.15±37.01% | 16.69±4.45% | 26.82±2.54% |
| Basidiomycota | 10.99±12.23% | 6.26±7.75% | 21.15±9.68% | 3.06±21.93% | 65.04±3.82% | 41.07±1.33% |
| unclassified | 2.15±1.09% | 0.13±0.09% | 1.39±1.48% | 0.19±5.25% | 7.21±3.36% | 15.29±1.77% |
| Mortierellomycota | 0.22±0.31% | 0.15±0.16% | 1.09±5.25% | 0.32±5.12% | 9.44±5.31% | 12.53±2.57% |
| Rozellomycota | 0.13±0.18% |  |  |  | 1.07±0.54% | 1.03±0.36% |
| Mucoromycota | 0.89±1.08% | 0.16%±0.12% | 0.04±0.04% | 0.29±1.20% | 0.50±0.37% | 3.25±1.46% |

HB and DB represent branches, HTU and DTU represent upper trunk, HTM and DTM represent middle trunk, HTL and DTL represent lower trunk, HTS and DTS represent surface soil, and HS and DS represent deep soil.

**Table S4.** Community composition at phylum level of bacteria in diseased trees and healthy trees

| Phylum | Diseased Trees | | | | | |
| --- | --- | --- | --- | --- | --- | --- |
|  | DB | DTU | DTM | DTL | DTS | DS |
| Proteobacteria | 69.01±5.50% | 88.44±1.46% | 64.02±13.49% | 50.73±27.66% | 47.72±1.60% | 43.54±9.16% |
| Cyanobacteria | 1.40±0.51% | 0.98±0.85% | 1.27±0.18% | 3.31±3.68% | 0.48±0.15% | 3.22±2.55% |
| Actinobacteria | 15.15±4.06% | 2.94±0.29% | 6.87±1.93% | 11.86±3.29% | 21.48±6.72% | 25.64±12.83% |
| Acidobacteria | 1.94±0.54% | 1.58±0.26% | 4.37±3.45% | 1.68±1.36% | 11.80±1.52% | 11.92±2.54% |
| Bacteroidetes | 10.25±2.98% | 5.04±1.25% | 8.57±3.12% | 10.78±10.08% | 3.29±0.57% | 1.72±0.73% |
| Firmicutes | 0.55±0.19% | 0.18±0.13% | 5.53±6.37% | 15.60±18.83% | 0.15±0.06% | 0.29±0.19% |
| Chloroflexi | 0.10±0.03% | 0.06±0.03% | 2.37±2.27% | 2.56±0.92% | 4.82±2.13% | 1.85±1.00% |
| Patescibacteria | 0.69±0.30% | 0.35±0.08% | 4.42±5.46% | 1.06±1.04% | 3.23±0.79% | 3.34±1.08% |
| WPS-2 | 0.02±0.02% | 0.01±0.02% | 0.01±0.02% | 0.22±0.11% | 2.57±0.63% | 3.27±1.27% |
| Verrucomicrobia | 0.44±0.10% | 0.25±0.16% | 0.44±0.46% | 0.21±0.14% | 1.41±0.31% | 1.67±0.76% |
| Planctomycetes | 0.04±0.02% | 0.03±0.01% | 0.23±0.16% | 0.16±0.11% | 1.33±0.35% | 1.60±0.83% |
| others | 0.40±0.18% | 0.11±0.05% | 1.90±1.49% | 1.84±0.40% | 1.71±0.53% | 1.92±0.62% |
| Phylum | Healthy Trees | | | | | |
|  | HB | HTU | HTM | HTL | HTS | HS |
| Proteobacteria | 2.31±0.80% | 57.17±7.91% | 67.14±10.42% | 57.22±16.34% | 39.44±2.36% | 32.28±3.91% |
| Cyanobacteria | 96.58±1.32% | 36.83±7.46% | 27.16±9.92% | 35.13±19.96% | 0.65±0.32% | 0.19±0.01% |
| Actinobacteria | 0.39±0.21% | 3.50±0.59% | 1.12±0.42% | 2.51±1.66% | 26.23±5.69% | 20.52±6.19% |
| Acidobacteria | 0.11±0.07% | 0.38±0.22% | 1.20±1.18% | 0.32±0.22% | 18.30±2.83% | 21.47±3.45% |
| Bacteroidetes | 0.17±0.05% | 0.56±0.10% | 1.22±0.85% | 0.88±0.36% | 2.25±0.31% | 1.06±0.16% |
| Firmicutes | 0.29±0.22% | 0.91±0.15% | 1.53±1.50% | 3.15±3.33% | 0.25±0.08% | 0.27±0.16% |
| Chloroflexi | 0.06±0.07% | 0.27±0.01% | 0.16±0.09% | 0.28±0.15% | 3.88±0.37% | 13.63±0.72% |
| Patescibacteria | 0.02±0.01% | 0.09±0.05% | 0.19±0.05% | 0.17±0.05% | 2.20±0.03% | 2.00±0.18% |
| WPS-2 | 0.01±0.00% | 0.05±0.01% | 0.06±0.06% | 0.02±0.02% | 2.56±0.93% | 3.37±0.21% |
| Verrucomicrobia | 0.01±0.01% | 0.01±0.01% | 0.03±0.02% | 0.03±0.02% | 1.40±0.19% | 1.73±0.43% |
| Planctomycetes | 0.01±0.01% | 0.05±0.02% | 0.05±0.02% | 0.08±0.08% | 1.15±0.17% | 1.35±0.45% |
| Others | 0.05±0.04% | 0.17±0.01% | 0.14±0.10% | 0.20±0.13% | 1.68±0.47% | 2.13±0.06% |

HB and DB represent branches, HTU and DTU represent upper trunk, HTM and DTM represent middle trunk, HTL and DTL represent lower trunk, HTS and DTS represent surface soil, and HS and DS represent deep soil. Others represent the numbers of partial phylum which the number < 1%.

**Table S5.** Community composition at class level of fungi in diseased trees and healthy trees

| Class | Diseased Trees | | | | | |
| --- | --- | --- | --- | --- | --- | --- |
|  | DB | DTU | DTM | DTL | DTS | DS |
| Sordariomycetes | 37.24±2.16% | 3.36±2.25% | 62.60±12.24% | 43.01±14.83% | 7.50±23.22% | 1.83±1.46% |
| Agaricomycetes | 0.16±0.16% | 33.28±36.46% | 0.06±0.08% | 10.21±3.07% | 28.86±19.19% | 11.60±7.13% |
| Tremellomycetes | 0.02±0.02% | 0.03±0.02% | 0.17±10.13% | 0.20±0.08% | 40.07±22.07% | 50.98±15.48% |
| Saccharomycetes | 11.87±4.10% | 45.98±26.54% | 34.74±12.11% | 35.19±8.66% | 0.05±20.04% | 0.02±0.02% |
| unclassified | 12.48±1.41% | 12.50±7.75% | 0.18±0.04% | 1.61±0.44% | 10.44±9.19% | 25.84±9.22% |
| Dothideomycetes | 37.34±5.83% | 3.62±3.05% | 1.75±0.46% | 0.59±0.49% | 0.88±12.55% | 0.97±1.19% |
| Eurotiomycetes | 0.20±0.14% | 0.28±0.19% | 0.30±0.32% | 8.74±8.18% | 5.49±4.24% | 3.34±2.69% |
| Mortierellomycetes |  | 0.01±0.01% | 0.00±0.00% | 0.03±0.04% | 3.24±1.34% | 1.44±1.05% |
| Others | 0.56±0.47% | 0.12±0.08% | 0.15±0.10% | 0.13±0.02% | 0.99±0.58% | 0.44±0.23% |
| Leotiomycetes | 0.13±0.08% | 0.82±0.25% | 0.04±0.01% | 0.30±0.16% | 2.47±1.56% | 3.54±2.36% |
| Class | Healthy Trees | | | | | |
|  | HB | HTU | HTM | HTL | HTS | HS |
| Sordariomycetes | 29.24±13.70% | 62.67±12.94% | 35.47±7.24% | 72.45±11.71% | 4.46±1.38% | 5.83±0.84% |
| Agaricomycetes | 0.11±0.09% | 0.33±0.30% | 0.49±0.30% | 0.21±0.05% | 54.23±7.48% | 18.56±11.37% |
| Tremellomycetes | 8.01±9.96% | 5.83±7.98% | 20.10±10.47% | 2.68±0.83% | 8.17±3.48% | 20.73±11.82% |
| Saccharomycetes | 0.77±0.50% | 1.21±0.66% | 10.47±3.12% | 1.81±0.71% | 0.01±0.01% | 0.02±0.01% |
| unclassified | 10.43±5.00% | 3.57±2.28% | 5.96±5.27% | 1.13±0.36% | 10.54±4.68% | 19.40±1.19% |
| Dothideomycetes | 28.59±3.91% | 9.40±10.26% | 11.04±3.29% | 4.17±0.95% | 2.76±1.01% | 1.47±0.14% |
| Eurotiomycetes | 16.71±5.47% | 16.40±2.37% | 12.77±4.92% | 16.65±11.30% | 6.34±1.95% | 11.62±1.70% |
| Mortierellomycetes | 0.22±0.31% | 0.15±0.16% | 1.09±1.44% | 0.32±0.38% | 9.43±5.31% | 12.48±2.55% |
| Others | 5.92±2.61% | 0.28±0.26% | 1.07±0.69% | 0.35±0.18% | 2.70±0.33% | 5.58±1.49% |
| Leotiomycetes | 0.00±0.00% | 0.15±0.06% | 1.54±2.00% | 0.23±0.09% | 1.37±0.58% | 4.31±1.73% |

HB and DB represent branches, HTU and DTU represent upper trunk, HTM and DTM represent middle trunk, HTL and DTL represent lower trunk, HTS and DTS represent surface soil, and HS and DS represent deep soil. Others represent the numbers of partial phylum which the number < 1%.

**Table S6.** Community composition at class level of bacteria in diseased trees and healthy trees

| Class | Diseased Trees | | | | | |
| --- | --- | --- | --- | --- | --- | --- |
|  | DB | DTU | DTM | DTL | DTS | DS |
| Gammaproteobacteria | 53.75±7.41% | 78.39±1.31% | 47.97±12.84% | 40.07±23.96% | 16.69±2.13% | 12.99±5.56% |
| Oxyphotobacteria | 1.39±0.51% | 0.98±0.84% | 1.22±0.13% | 3.27±3.65% | 0.03±0.00% | 0.04±0.01% |
| Alphaproteobacteria | 14.52±1.19% | 9.94±1.39% | 14.14±3.70% | 9.95±5.54% | 28.54±0.85% | 26.60±5.54% |
| Actinobacteria | 15.15±4.06% | 2.94±0.29% | 6.87±1.93% | 11.86±3.29% | 21.48±6.72% | 24.30±12.68% |
| Acidobacteriia | 1.86±0.62% | 1.53±0.23% | 2.09±0.57% | 0.97±0.89% | 11.19±1.25% | 12.76±3.84% |
| Bacteroidia | 10.24±2.98% | 5.04±1.26% | 8.56±3.10% | 10.64±10.15% | 3.20±0.50% | 1.60±0.81% |
| Bacilli | 0.36±0.08% | 0.12±0.07% | 2.48±2.26% | 11.33±14.56% | 0.14±0.06% | 0.21±0.13% |
| Saccharimonadia | 0.67±0.30% | 0.34±0.07% | 4.33±5.40% | 0.98±1.04% | 2.69±0.67% | 1.90±0.35% |
| Deltaproteobacteria | 0.74±0.79% | 0.11±0.04% | 1.87±2.14% | 0.69±0.32% | 2.47±0.38% | 2.66±0.71% |
| Ktedonobacteria | 0.00±0.00% | 0.01±0.00% | 0.17±0.18% | 0.06±0.04% | 3.81±1.61% | 3.18±3.32% |
| norank | 0.02±0.02% | 0.01±0.02% | 0.01±0.02% | 0.22±0.11% | 2.57±0.63% | 2.77±0.90% |
| Clostridia | 0.15±0.11% | 0.06±0.06% | 2.60±3.54% | 3.81±3.80% | 0.01±0.00% | 0.06±0.05% |
| Verrucomicrobiae | 0.44±0.10% | 0.25±0.16% | 0.44±0.46% | 0.21±0.14% | 1.41±0.31% | 1.69±0.77% |
| AD3 | 0.00±0.01% | 0.00±0.00% | 0.01±0.02% | 0.03±0.04% | 0.34±0.18% | 1.85±2.50% |
| Planctomycetacia | 0.02±0.01% | 0.03±0.01% | 0.08±0.10% | 0.08±0.06% | 1.27±0.36% | 1.19±0.42% |
| Gemmatimonadetes | 0.04±0.05% | 0.01±0.02% | 0.70±0.91% | 0.59±0.24% | 0.68±0.34% | 0.55±0.19% |
| Subgroup_6 | 0.02±0.02% | 0.01±0.01% | 1.60±2.12% | 0.19±0.18% | 0.39±0.36% | 0.16±0.13% |
| Melainabacteria | 0.00±0.00% | 0.00±0.00% | 0.05±0.06% | 0.02±0.03% | 0.44±0.15% | 2.28±2.95% |
| Anaerolineae | 0.02±0.02% | 0.02±0.01% | 0.44±0.30% | 1.01±0.35% | 0.23±0.22% | 0.13±0.17% |
| Chloroflexia | 0.04±0.01% | 0.03±0.01% | 0.62±0.64% | 1.13±0.70% | 0.06±0.03% | 0.05±0.07% |
| Others | 0.55±0.21% | 0.18±0.12% | 3.73±2.98% | 2.89±1.18% | 2.36±0.56% | 3.03±1.64% |
| Class | Healthy Trees | | | | | |
|  | HB | HTU | HTM | HTL | HTS | HS |
| Gammaproteobacteria | 1.27±0.28% | 54.45±7.34% | 59.38±8.41% | 54.13±14.90% | 10.97±0.81% | 10.81±2.34% |
| Oxyphotobacteria | 96.58±1.32% | 36.82±7.47% | 27.14±9.91% | 35.12±19.97% | 0.04±0.01% | 0.03±0.00% |
| Alphaproteobacteria | 1.00±0.59% | 2.53±0.77% | 7.66±3.51% | 2.94±1.35% | 26.21±2.80% | 19.72±1.58% |
| Actinobacteria | 0.39±0.21% | 3.50±0.59% | 1.12±0.42% | 2.51±1.66% | 26.23±5.69% | 20.52±6.19% |
| Acidobacteriia | 0.08±0.05% | 0.34±0.22% | 1.10±1.22% | 0.20±0.18% | 17.16±2.95% | 21.00±3.47% |
| Bacteroidia | 0.17±0.05% | 0.51±0.07% | 1.22±0.85% | 0.87±0.35% | 2.22±0.31% | 0.98±0.11% |
| Bacilli | 0.23±0.20% | 0.48±0.04% | 1.30±1.46% | 2.90±3.24% | 0.24±0.07% | 0.21±0.11% |
| Saccharimonadia | 0.01±0.00% | 0.07±0.04% | 0.14±0.08% | 0.12±0.09% | 1.70±0.05% | 1.70±0.17% |
| Deltaproteobacteria | 0.04±0.01% | 0.19±0.07% | 0.11±0.03% | 0.16±0.09% | 2.25±0.51% | 1.75±0.06% |
| Ktedonobacteria | 0.00±0.00% | 0.05±0.03% | 0.05±0.03% | 0.03±0.03% | 2.39±0.58% | 5.82±1.73% |
| norank | 0.01±0.00% | 0.05±0.01% | 0.06±0.03% | 0.02±0.02% | 2.56±0.93% | 3.37±0.21% |
| Clostridia | 0.04±0.02% | 0.36±0.11% | 0.19±0.06% | 0.23±0.16% | 0.01±0.01% | 0.05±0.05% |
| Verrucomicrobiae | 0.01±0.01% | 0.01±0.01% | 0.03±0.02% | 0.03±0.02% | 1.40±0.19% | 1.73±0.43% |
| AD3 | 0.00±0.00% | 0.00±0.00% | 0.00±0.00% |  | 0.67±0.21% | 6.47±1.04% |
| Planctomycetacia | 0.00±0.01% | 0.03±0.01% | 0.04±0.03% | 0.04±0.03% | 1.08±0.21% | 1.33±0.44% |
| Gemmatimonadetes | 0.01±0.01% | 0.00±0.00% | 0.03±0.04% | 0.05±0.02% | 0.62±0.11% | 0.77±0.03% |
| Subgroup_6 | 0.01±0.01% | 0.03±0.01% | 0.04±0.05% | 0.03±0.01% | 0.37±0.15% | 0.35±0.04% |
| Melainabacteria |  | 0.01±0.01% | 0.01±0.01% | 0.01±0.01% | 0.59±0.31% | 0.15±0.02% |
| Anaerolineae | 0.03±0.04% | 0.09±0.06% | 0.04±0.04% | 0.11±0.11% | 0.20±0.03% | 0.47±0.09% |
| Chloroflexia | 0.00±0.01% | 0.02±0.02% | 0.03±0.01% | 0.04±0.03% | 0.09±0.01% | 0.13±0.03% |
| Others | 0.11±0.06% | 0.46±0.10% | 0.31±0.15% | 0.47±0.30% | 2.99±0.33% | 2.63±0.09% |

HB and DB represent branches, HTU and DTU represent upper trunk, HTM and DTM represent middle trunk, HTL and DTL represent lower trunk, HTS and DTS represent surface soil, and HS and DS represent deep soil. Others represent the numbers of partial phylum which the number < 1%.

**Table S7.** The list of the top 10 (>1%) OTU of fungi showing significant differences in the abundance between healthy (H) and diseased (D) trees.

| Site | OTUs | Taxonomy | p-Value | Abundance Pattern |
| --- | --- | --- | --- | --- |
|  | OTU1704 | *Diplodia* | 0.0007881 | HB<DB |
|  | OTU1738 | *Graphilbum* | 0.0009128 | HB<DB |
|  | OTU100 | *Pestalotiopsis* | 0.00117 | HB>DB |
|  | OTU1915 | Saccharomycetales | 0.001916 | HB<DB |
| Branch | OTU272 | *Penicillium* | 0.003004 | HB>DB |
|  | OTU344 | *Cladosporium* | 0.001863 | HB>DB |
|  | OTU51 | *Devriesia* | 0.009496 | HB>DB |
|  | OTU261 | Nectriaceae | 0.04367 | HB>DB |
|  | OTU120 | *Wallemia* | 0.03678 | HB>DB |
|  | OTU128 | *Capnobotryella* | 0.003202 | HB>DB |
|  | OTU1800 | Ganodermataceae | 0.01263 | HTU<DTU |
|  | OTU1772 | *Candida* | 0.001445 | HTU<DTU |
|  | OTU255 | *Trichoderma* | 0.0007982 | HTU>DTU |
|  | OTU580 | *Trichoderma* | 0.0007675 | HTU>DTU |
| Trunk | OTU1821 | Saccharomycetales | 0.001022 | HTU<DTU |
| upper | OTU159 | *Talaromyces* | 0.0004248 | HTU>DTU |
|  | OTU160 | *Fusarium* | 0.006007 | HTU>DTU |
|  | OTU148 | *Arthrographis* | 0.004906 | HTU>DTU |
|  | OTU171 | *Trichoderma* | 0.002903 | HTU>DTU |
|  | OTU1704 | *Diplodia* | 0.0007881 | HTU<DTU |
|  | OTU1857 | *Graphilbum* | 0.001052 | HTM<DTM |
|  | OTU1861 | Xylariales | 0.001933 | HTM<DTM |
|  | OTU1915 | Saccharomycetales | 0.001916 | HTM<DTM |
|  | OTU1821 | Saccharomycetales | 0.001022 | HTM<DTM |
| Trunk | OTU1851 | *Xenoacremonium* | 0.001275 | HTM<DTM |
| middle | OTU1772 | *Candida* | 0.001445 | HTM>DTM |
|  | OTU255 | *Trichoderma* | 0.0007982 | HTM>DTM |
|  | OTU406 | *Penicillium* | 0.02743 | HTM>DTM |
|  | OTU261 | Nectriaceae | 0.04367 | HTM>DTM |
|  | OTU706 | *Alternaria* | 0.0112 | HTM>DTM |
|  | OTU421 | *Fusarium* | 0.01193 | HTL>DTL |
|  | OTU1738 | *Graphilbum* | 0.0009128 | HTL<DTL |
|  | OTU1915 | Saccharomycetales | 0.001916 | HTL<DTL |
|  | OTU255 | *Trichoderma* | 0.0007982 | HTL>DTL |
| Trunk | OTU1821 | Saccharomycetales | 0.001022 | HTL<DTL |
| lower | OTU1922 | *Stereum* | 0.001933 | HTL<DTL |
|  | OTU1878 | *Trichoderma* | 0.003839 | HTL<DTL |
|  | OTU580 | *Trichoderma* | 0.0007675 | HTL>DTL |
|  | OTU2489 | *Trichoderma* | 0.004849 | HTL>DTL |
|  | OTU419 | *Fusarium* | 0.004363 | HTL>DTL |
|  | OTU2785 | *Saitozyma* | 0.0009707 | HTS<DTS |
|  | OTU645 | *Russula* | 0.0007048 | HTS>DTS |
|  | OTU1939 | *Membranomyces* | 0.0008008 | HTS<DTS |
| Top | OTU849 | *Russula* | 0.001933 | HTS>DTS |
| soil | OTU1011 | *Mortierella* | 0.0007537 | HTS>DTS |
|  | OTU1136 | *Russula* | 0.0008008 | HTS>DTS |
|  | OTU2266 | Sordariomycetes | 0.0005226 | HTS<DTS |
|  | OTU2611 | Thelephoraceae | 0.0005234 | HTS<DTS |
|  | OTU1201 | *Penicillium* | 0.002017 | HTS<DTS |
|  | OTU2388 | *Trechispora* | 0.002181 | HTS<DTS |
|  | OTU2785 | *Saitozyma* | 0.0009707 | HS<DS |
|  | OTU645 | *Russula* | 0.0007048 | HS>DS |
|  | OTU1011 | *Mortierella* | 0.0007537 | HS>DS |
|  | OTU2621 | Basidiomycota | 0.0007366 | HS<DS |
| Deep | OTU2611 | Thelephoraceae | 0.0005234 | HS<DS |
| soil | OTU2528 | Rozellomycota | 0.0004829 | HS<DS |
|  | OTU1201 | *Penicillium* | 0.002017 | HS>DS |
|  | OTU1939 | *Membranomyces* | 0.0008008 | HS<DS |
|  | OTU580 | *Trichoderma* | 0.0007675 | HS>DS |
|  | OTU965 | *Mortierella* | 0.0002932 | HS>DS |

HB and DB represent branches, HTU and DTU represent upper trunk, HTM and DTM represent middle trunk, HTL and DTL represent lower trunk, HTS and DTS represent surface soil, and HS and DS represent deep soil.

**Table S8.** The list of the top 10 (>1%) OTU of bacteria showing significant differences in the abundance between healthy (H) and diseased (D) trees.

| Site | OTUs | Taxonomy | p-Value | Abundance Pattern |
| --- | --- | --- | --- | --- |
|  | OTU711 | Chloroplast | 0.0003449 | HB>DB |
|  | OTU4132 | *Dyella* | 0.03482 | HB<DB |
|  | OTU2682 | *Curtobacterium* | 0.007867 | HB<DB |
|  | OTU2772 | *Dyella* | 0.02816 | HB<DB |
| Branch | OTU2448 | *Sphingomonas* | 0.01967 | HB<DB |
|  | OTU5162 | *Erwinia* | 0.01858 | HB<DB |
|  | OTU2455 | *Gryllotalpicola* | 0.01198 | HB<DB |
|  | OTU2757 | Chitinophagaceae | 0.02621 | HB<DB |
|  | OTU5006 | Geminicoccaceae | 0.00375 | HB<DB |
|  | OTU2786 | Burkholderiaceae | 0.007416 | HB<DB |
|  | OTU711 | Chloroplast | 0.01927 | HTU>DTU |
|  | OTU538 | *Pantoea* | 0.002082 | HTU<DTU |
|  | OTU2791 | *Pantoea* | 0.00195 | HTU<DTU |
|  | OTU759 | *Enterobacter* | 0.002678 | HTU>DTU |
| Trunk | OTU5162 | *Erwinia* | 0.04051 | HTU<DTU |
| upper | OTU310 | *Pseudoxanthomonas* | 0.01002 | HTU<DTU |
|  | OTU327 | Burkholderiaceae | 0.04413 | HTU<DTU |
|  | OTU2794 | Burkholderiaceae | 0.003952 | HTU<DTU |
|  | OTU443 | *Luteibacter* | 0.02086 | HTU<DTU |
|  | OTU2448 | *Sphingomonas* | 0.0229 | HTU<DTU |
|  | OTU310 | *Pseudoxanthomonas* | 0.02941 | HTM<DTM |
|  | OTU538 | *Pantoea* | 0.03783 | HTM>DTM |
|  | OTU759 | *Enterobacter* | 0.04821 | HTM>DTM |
|  | OTU2794 | Burkholderiaceae | 0.04532 | HTM<DTM |
| Trunk | OTU443 | *Luteibacter* | 0.0452 | HTM<DTM |
| middle | OTU564 | Burkholderiaceae | 0.0003265 | HTM>DTM |
|  | OTU2791 | *Pantoea* | 0.01997 | HTM>DTM |
|  | OTU798 | *Pseudomonas* | 0.01112 | HTM>DTM |
|  | OTU813 | *Silvimonas* | 0.01924 | HTM>DTM |
|  | OTU3630 | *Serratia* | 0.01808 | HTM<DTM |
|  | OTU759 | *Enterobacter* | 0.001577 | HTL>DTL |
|  | OTU2791 | *Pantoea* | 0.02139 | HTL>DTL |
|  | OTU690 | *Massilia* | 0.009116 | HTL>DTL |
|  | OTU4777 | *Defluviicoccus* | 0.01378 | HTL<DTL |
| Trunk | OTU3629 | *Ilumatobacter* | 0.01295 | HTL<DTL |
| lower | OTU581 | Chloroplast | 0.0171 | HTL>DTL |
|  | OTU2692 | Burkholderiaceae | 0.00704 | HTL<DTL |
|  | OTU4377 | *Luteitalea* | 0.003932 | HTL<DTL |
|  | OTU4895 | Thermomicrobiales | 0.0411 | HTL<DTL |
|  | OTU5839 | Burkholderiaceae | 0.0405 | HTL<DTL |
|  | OTU2303 | Xanthobacteraceae | 0.04638 | HTS>DTS |
|  | OTU327 | Burkholderiaceae | 0.03218 | HTS<DTS |
|  | OTU1406 | Xanthobacteraceae | 0.02851 | HTS>DTS |
| Top | OTU6417 | *Acidothermus* | 0.03543 | HTS>DTS |
| soil | OTU2794 | Burkholderiaceae | 0.02451 | HTS<DTS |
|  | OTU5406 | *Acidothermus* | 0.04465 | HTS>DTS |
|  | OTU5762 | *Mycobacterium* | 0.0237 | HTS>DTS |
|  | OTU1970 | Xiphinematobacteraceae | 0.04115 | HTS>DTS |
|  | OTU5376 | Saccharimonadales | 0.04336 | HTS<DTS |
|  | OTU1040 | *Acidothermus* | 0.02705 | HTS>DTS |
|  | OTU1968 | Acidobacteriia | 0.01944 | HS>DS |
|  | OTU1655 | Acidobacteriia | 0.03504 | HS>DS |
|  | OTU2229 | AD3 | 0.02127 | HS>DS |
|  | OTU6105 | *Roseiarcus* | 0.04905 | HS<DS |
| Deep | OTU5418 | Actinobacteria | 0.02301 | HS>DS |
| soil | OTU485 | Caulobacteraceae | 0.0002475 | HS<DS |
|  | OTU957 | Acidobacteriales | 0.02455 | HS>DS |
|  | OTU1707 | AD3 | 0.04707 | HS>DS |
|  | OTU2002 | *Acidibacter* | 0.01993 | HS>DS |
|  | OTU2257 | Gaiellales | 0.006467 | HS>DS |

HB and DB represent branches, HTU and DTU represent upper trunk, HTM and DTM represent middle trunk, HTL and DTL represent lower trunk, HTS and DTS represent surface soil, and HS and DS represent deep soil.
